# Systematic review and narrative synthesis of computerized audit and feedback systems in healthcare

**DOI:** 10.1093/jamia/ocac031

**Published:** 2022-03-10

**Authors:** Jung Yin Tsang, Niels Peek, Iain Buchan, Sabine N van der Veer, Benjamin Brown

**Affiliations:** Centre for Health Informatics, Division of Informatics, Imaging and Data Science, Faculty of Biology, Medicine and Health, Manchester Academic Health Science Centre, The University of Manchester, Manchester, UK; Centre for Primary Care and Health Services Research, University of Manchester, Manchester, UK; NIHR Greater Manchester Patient Safety Translational Research Centre (GMPSTRC), University of Manchester, Manchester, UK; Centre for Health Informatics, Division of Informatics, Imaging and Data Science, Faculty of Biology, Medicine and Health, Manchester Academic Health Science Centre, The University of Manchester, Manchester, UK; NIHR Greater Manchester Patient Safety Translational Research Centre (GMPSTRC), University of Manchester, Manchester, UK; NIHR Applied Research Collaboration Greater Manchester, University of Manchester, Manchester, UK; Institute of Population Health, University of Liverpool, Liverpool, UK; Centre for Health Informatics, Division of Informatics, Imaging and Data Science, Faculty of Biology, Medicine and Health, Manchester Academic Health Science Centre, The University of Manchester, Manchester, UK; Centre for Health Informatics, Division of Informatics, Imaging and Data Science, Faculty of Biology, Medicine and Health, Manchester Academic Health Science Centre, The University of Manchester, Manchester, UK; Centre for Primary Care and Health Services Research, University of Manchester, Manchester, UK; NIHR Greater Manchester Patient Safety Translational Research Centre (GMPSTRC), University of Manchester, Manchester, UK

**Keywords:** clinical audit, feedback, quality improvement, benchmarking, informatics, systematic review

## Abstract

**Objectives:**

(1) Systematically review the literature on computerized audit and feedback (e-A&F) systems in healthcare. (2) Compare features of current systems against e-A&F best practices. (3) Generate hypotheses on how e-A&F systems may impact patient care and outcomes.

**Methods:**

We searched MEDLINE (Ovid), EMBASE (Ovid), and CINAHL (Ebsco) databases to December 31, 2020. Two reviewers independently performed selection, extraction, and quality appraisal (Mixed Methods Appraisal Tool). System features were compared with 18 best practices derived from Clinical Performance Feedback Intervention Theory. We then used realist concepts to generate hypotheses on mechanisms of e-A&F impact. Results are reported in accordance with the PRISMA statement.

**Results:**

Our search yielded 4301 unique articles. We included 88 studies evaluating 65 e-A&F systems, spanning a diverse range of clinical areas, including medical, surgical, general practice, etc. Systems adopted a median of 8 best practices (interquartile range 6–10), with 32 systems providing near real-time feedback data and 20 systems incorporating action planning. High-confidence hypotheses suggested that favorable e-A&F systems prompted specific actions, particularly enabled by timely and role-specific feedback (including patient lists and individual performance data) and embedded action plans, in order to improve system usage, care quality, and patient outcomes.

**Conclusions:**

e-A&F systems continue to be developed for many clinical applications. Yet, several systems still lack basic features recommended by best practice, such as timely feedback and action planning. Systems should focus on actionability, by providing real-time data for feedback that is specific to user roles, with embedded action plans.

**Protocol Registration:**

PROSPERO CRD42016048695.

## INTRODUCTION

Audit and feedback (A&F) is widely used to improve care quality and health outcomes.^1^ Through summarizing clinical performance over time (audit), and presenting this information to health professionals and their organizations (feedback), it can drive improvements in health outcomes.^1–3^ There is established literature on predictors of A&F effectiveness, such as targeting low baselines, delivering feedback through supervisors, and frequent feedback.^1^^,^^3^^,^^4^ This has led to theories of how A&F produces change in clinical practice and hypothesized features of best practice.^5^^,^^6^ We previously developed a clinical performance feedback intervention theory (CP-FIT): a framework for A&F interventions describing how feedback works and factors that influence success.^5^ However, little is known about to what extent this translates to automated or computerized forms of A&F using digital care records and computational approaches, which are becoming increasingly adopted.

Computerized or electronic audit and feedback (e-A&F) systems, often delivered as “dashboards,” generally incorporate visualization elements to deliver feedback of clinical performance.^7^ With increasing availability of linked care record data, they offer potential advantages over manual A&F methods through lower costs of producing the audits and quicker feedback.^7^ Developing e-A&F systems have also changed the dynamics of how clinical performance is understood, evolving from single graphical displays requiring human assistance for feedback, into automated multi-functional feedback displays with interactive components.^8^ Over the last decade, e-A&F systems have moved away from static reports, as interactive interfaces enable users to “drill down,” filter and prioritize the data, carrying greater potential for flexibility and specificity in feedback.^1^ E-A&F systems are generally used away from the point-of-care (unlike clinical decision support tools), but can produce timely improvements on individual, team, or organizational levels depending on how feedback data is used to review care performance.^7^

Two previous systematic reviews examining e-A&F, yielded limited insights into the characteristics of successful systems due to the heterogeneity of studies and inclusion criteria.^7^^,^^9^ The most recent (2017) review focused on behavior change theory and included only 7 randomized controlled trials (RCTs).^7^ This needed updating and extending to consider a wider range of current e-A&F systems in more detail.

A&F systems continue to demonstrate highly variable effects on patient care, though effect sizes have been plateauing for some time.^4^ Rather than simply studying outcomes, a greater focus on optimization of intervention design is required.^10^ There is a need for more comprehensive evidence of e-A&F that considers and extends best practice theory to define successful features and components of these systems.^7^^,^^9^ Previous studies have shown that contextual factors need to be considered, which directly affect e-A&F implementation, such as data infrastructure and existing ways of working.^7^^,^^9^^,^^11^ A narrative synthesis allows deeper exploration of intervention components, contextual factors, and mechanisms of action to generate further hypotheses regarding outcomes and effect modifiers.^12^

The aim of this study was to summarize and evaluate the current state of e-A&F, synthesizing the literature to provide useful evidence through learning from successes and failures. Using an extended theoretical framework, we explored how e-A&F system design may be optimized to reduce variability in outcomes.

## OBJECTIVES

Objective 1: Systematically review and summarize the literature on published e-A&F systems in healthcare.

Objective 2: Compare features of these e-A&F systems against generic A&F best practices.

Objective 3: Generate hypotheses on how e-A&F systems may impact patient care and outcomes

## METHODS

This article is consistent with PRISMA standards for systematic reviews.^13^ The protocol of our study is published on the International Prospective Register of Systematic Reviews [PROSPERO CRD42016048695].

### Search strategy

We replicated the search strategy of the latest Cochrane review on A&F.^1^ The search terms for RCT filters were replaced with those relating to computerization (Supplementary File S1), based on the scoping search (described in our protocol) and previous literature.^1^^,^^5^ We searched MEDLINE (Ovid), EMBASE (Ovid), and CINAHL (Ebsco) databases starting from January 1 1999, based on the earliest publication date of papers from our scoping searches, up to December 31, 2020. For each included article, we performed a supplementary search (undertaken up to January 31, 2021) that consisted of reference list, citation, and related article searching to identify further relevant articles. Related article and citation searching was performed in Google Scholar and limited to the first 100 articles to maintain relevance.

### Study selection and data extraction

The inclusion criteria are presented in Table 1. We included all peer-reviewed studies on interactive e-A&F systems used by health professionals for care improvements that were implemented in clinical practice. Two reviewers (JT and BB) independently screened titles and abstracts using the inclusion criteria. Citations that were deemed relevant by either reviewer had full texts obtained. All full manuscripts were then independently read by the 2 reviewers, and the inclusion criteria reapplied with any disagreements being resolved through discussion. Data extraction and quality appraisal (see below) were undertaken concurrently using a standardized data extraction tool (Supplementary File S2) by JT and reviewed independently by a second researcher (BB). Further discussion of the data and resolving of discrepancies occurred at weekly meetings. Data were collected regarding studies’ characteristics, outcomes, and features of the e-A&F system being studied.

**Table 1.** Inclusion criteria and typical examples of exclusions

Inclusion criteria	Typical exclusion examples
Population	
The system is primarily intended for use by healthcare professionals (including clinicians and nonclinicians eg, managers)	Websites primarily intended to help patients choose healthcare provider
Intervention	
The system provides clinical performance feedback to healthcare professionals. Clinical performance includes compliance with pre-defined clinical standards, as well as patient outcomes	Systems that provide feedback primarily regarding nonclinical performance, for example, care costs, patient access, and epidemiological surveillance
Clinical performance data are obtained from medical records, computerized databases, or observations from patients	Clinical performance feedback systems based on peer or supervisor observation
Feedback relates to multiple patients	Highly specific systems that only provide data for a single patient
Feedback to inform quality improvement actions at individual, team, or organizational levels	Intensive care unit dashboards that summarize patients’ current clinical status to primarily inform bedside or point-of care decisions
Feedback is provided via a dynamic interface with which the user can interact, (eg, a web-based portal or desktop application)	Feedback primarily provided on paper, verbally or via static interfaces such as screensavers, e-mail, or electronic documents
Providing clinical performance feedback is a core and essential function of the system, that is, in systems with additional functionalities, it is unlikely these would be offered in the absence of such feedback	Point-of-care reminder systems that additionally provide clinical performance feedback once per year
Outcome	
The system primarily aims to improve clinical performance (as defined above)	Systems primarily intended to reduce costs
Study type	
Empirical research evaluation studies of systems being used by healthcare professionals as target end-users, reporting findings from primary data collection and analysis (either qualitative or quantitative) focusing on the behavior of end-users using the system, outcomes of their behavior from using the system, or performance of the system	Articles reporting system descriptions, or studies conducted with members or the system development or research team
Peer-reviewed publications in scholarly journals, written in English with abstracts available for review	Conference abstracts, theses, gray literature, and non-English literature

### Quality appraisal

We performed quality appraisal (risk of bias) using the Mixed Methods Appraisal Tool (MMAT) version 2011.^14^ The MMAT is a validated tool that includes assessment criteria of methodological quality for quantitative, qualitative, and mixed methods studies.^14^^,^^15^ These criteria include 2 screening questions and 3–4 design-specific questions, with different study designs having different quality criteria. The results are presented as 1–4 stars, allowing direct comparison between different study types. This was incorporated into a GRADE-CERQual assessment to explicitly evaluate the confidence placed in each individual set of findings from objective 3 (see below).^16^ The GRADE-CERQuaL approach incorporates 4 components including methodological limitations, relevance to the review question, coherence of the finding, and adequacy of data. Ratings of “high,” “moderate,” or “low” confidence were given through considering these 4 components in the context of reviewing the evidence supporting the findings, and its relation to the wider review question. Thus, quality appraisal was used to inform data synthesis rather than determine study inclusion to avoid excluding “low quality” studies that still generated valuable insights.^17^

### Analysis and synthesis

CP-FIT took a central role in framing the analysis and synthesis of data.^5^ CP-FIT builds on 30 pre-existing theories from a range of disciplines including behavior change, goal setting, context, psychological, sociological, and technology theories.^5^ It outlines factors for successful feedback cycles in producing behavior changes in health professionals.^5^ To achieve each of our objectives, we undertook the following analyses:

#### Objective 1: systematically review the literature on e-A&F systems in healthcare

We categorized common conceptual domains and dimensions of e-A&F systems, allowing grouping and contrasting of interventions to supplement further analyses. Using thematic analysis, we developed codes that described and categorized different features of the e-A&F systems.^18^ Codes were created both inductively from the data, and by deductively applying codes that describe A&F systems taken from CP-FIT.^5^

#### Objective 2: compare features of e-A&F systems against generic A&F “best practices”

We compared each e-A&F system to a list of features from current literature thought to be associated with effective A&F, determining whether each feature was present, absent, or not-reported.^1^^,^^5^^,^^6^ We focused on 18 effective features that could be measured more objectively included those from the latest Cochrane review, in addition to theorized features within CP-FIT.^1^^,^^5^ These included a list of defined “cointerventions,” such as “clinical education” and “financial rewards,” but more subjective features of best practice such as credibility and adaptability were excluded.^5^ We assumed that existing ‘best practices’ for A&F would be applicable to e-A&F systems, but also looked to refine these best practices to increase their relevance to e-A&F. We used linear regression to estimate the trend of best practice features adopted over time.

#### Objective 3: generate hypotheses on how e-A&F systems may impact patient care and outcomes

We adopted realist concepts to summarize our findings and to explore features of e-A&F systems as interventions implemented within complex health and social contexts.^5^^,^^19^ Moving beyond traditional review methods, realist methodology allowed us to look past overall successes or failures of e-A&F systems to generate explanations about how and why these systems work, for whom, and in what contexts.^19^ Drawing on findings developed in objectives 1 and 2, descriptive and analytical themes were organized into intervention-context-mechanism-outcome (ICMO) configurations to generate further hypotheses.^19^^,^^20^ The resulting synthesis highlighted possible intervention factors (I) of e-A&F systems that when implemented in a specific context (C), acted through various mechanisms (M) to produce particular outcomes (O) of interest (including usage, care quality, and patient outcomes). As in CP-FIT, mechanisms (M) were defined as underlying explanations of how and why an intervention works, related to the feedback itself, the recipient, and the wider context.^5^^,^^19^ Each ICMO configuration was assessed through GRADE-CERQual to explicitly evaluate our confidence for each hypothesis. Included papers were then reread to iteratively test and refine our emerging hypotheses, starting with papers with higher scores of the quality appraisal and GRADE-CERQual.^19^

## RESULTS

### Study selection

**Figure 1.**
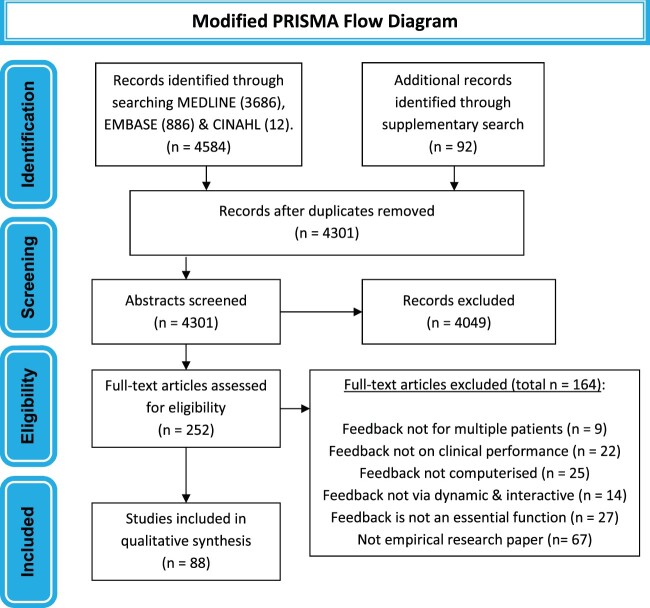
Flow diagram summarizing study selection process. Illustration of the steps used in the study selection process.

The search of the 3 databases yielded 4584 articles, with 92 more articles being identified in the supplementary search (Figure 1). After removing duplicates, 4301 abstracts were screened. Most articles removed at this stage did not describe an e-A&F system impacting clinical care. A total of 252 full-text articles were assessed and 88 papers studying 65 systems were included in total.

### Systematic review of published e-A&F systems (Objective 1)

Included studies varied in study type, timeframe, and reporting of results, with some studies looking at clinician performance, others looking at outcome measures, and some examining systems utilization and integration.^21–108^ The main characteristics are summarized in Table 2 with full details in Supplementary File S3.

**Table 2.** Frequency of main study characteristics

		Count (%)^a^
Publication year	2016–2020	43 (49%)
	2011–2015	34 (39%)
	2005–2010	11 (12%)
Quality appraisal (4* being lowest risk of bias)	4*	4 (4%)
	3*	37 (42%)
	2*	33 (38%)
	1*	14 (16%)
Study type	Randomized controlled trial	21 (24%)
	Nonrandomized controlled trial	3 (3%)
	Cohort study	5 (6%)
	Before and after study	8 (9%)
	Cross sectional study	3 (3%)
	Other quantitative study	11 (12%)
	Qualitative study	27 (31%)
	Mixed methods study	10 (11%)
Continent	North America	57 (65%)
	Europe	26 (30%)
	Asia	4 (4%)
	Australia	1 (1%)
Setting	Hospital care (including secondary and tertiary settings)	51 (58%)
	Outpatient care (including specialty and primary care settings)	36 (41%)
	Nursing home	1 (1%)
Specialty area	Medication safety	19 (22%)
	Diabetes	17 (19%)
	Cardiovascular	15 (17%)
	Respiratory	6 (7%)
	Oncology	9 (10%)
	Nephrology	2 (2%)
	Geriatrics	4 (4%)
	General medicine	4 (4%)
	Infectious disease	11 (12%)
	Surgery	5 (6%)
	Obstetrics	1 (1%)
	Pediatrics	3 (3%)
	Radiology	4 (4%)
	Psychiatry (including substance misuse)	5 (6%)

a Counts may add to more than 100% where papers are in multiple categories.

A summary of e-A&F system features is presented in Table 3. Systems targeted a diverse range of aspects of care, the most common being prescribing (32 out of 65 systems) and chronic disease management (24 systems). Most systems (57 of 65) were used by doctors, with 29 systems being designed for doctors alone and 21 systems also involving users with managerial or senior leadership roles. For feedback display, over 70% of systems (46 of 65) included graphical elements. These systems varied in their presentation of line, bar, pie, and box and whisker plots, with some systems (27 of 65) presenting more than one type of graph. Over 80% (53 of 65) systems incorporated benchmarking elements with a similar number of systems (51 of 65) displaying specific performance data at individual or practice level. About two-thirds (43 of 65) provided lists of patients, with over a third (24 of 65) providing detailed patient-level data. Over half (34 of 65) deployed interactive functions for prioritization including sorting and color coding functions.

**Table 3.** Summary of computerized audit and feedback (e-A&F) system features

**Goal** *What aspect(s) of clinical care were targeted?*	Prescribing^27^^,^^28^^,^^32^^,^^35^^,^^37^^,^^48^^,^^50^^,^^53^^,^^55–57^^,^^61^^,^^62^^,^^65^^,^^67^^,^^69^^,^^72^^,^^74^^,^^75^^,^^77^^,^^79^^,^^81^^,^^84^^,^^85^^,^^89^^,^^93–95^^,^^98^^,^^100^^,^^102^^,^^103^
	Blood test use and monitoring^22^^,^^39^^,^^55^^,^^63^^,^^80^^,^^81^
	Skill-based performance (eg, surgical/radiological)^24^^,^^31^^,^^40^^,^^42^^,^^51^^,^^96^^,^^99^^,^^107^
	Chronic disease management^21^^,^^26^^,^^32^^,^^33^^,^^35^^,^^37^^,^^39^^,^^43^^,^^48^^,^^54–56^^,^^61^^,^^69^^,^^75^^,^^77–79^^,^^84^^,^^91^^,^^93^^,^^97^^,^^101^^,^^103^^,^^104^
	Acute condition management^22^^,^^37^^,^^41^^,^^53^^,^^67^^,^^70^^,^^86^^,^^92^
	Disease prevention and screening^25^^,^^27^^,^^35^^,^^39^^,^^54^^,^^55^^,^^60^^,^^61^^,^^71^^,^^74^^,^^75^^,^^79^^,^^101^^,^^103^^,^^104^
	Nursing care^40^^,^^52^^,^^59^^,^^73^^,^^75^
	Discharge care^21^^,^^80^
	Patient experience^25^^,^^51^^,^^103^
**Health professional** *What were the professional role(s) of the users?*	Doctors only^24–26^^,^^31–33^^,^^37^^,^^42^^,^^48^^,^^60–63^^,^^69^^,^^71^^,^^74^^,^^79^^,^^80^^,^^84^^,^^89^^,^^92^^,^^93^^,^^95–97^^,^^99^^,^^101^^,^^103^^,^^104^
	Doctors and nurses^27^^,^^40^^,^^41^^,^^51^^,^^54^^,^^67^^,^^78^^,^^91^^,^^105^
	Doctors and pharmacists^28^^,^^57^^,^^65^^,^^81^^,^^98^
	Doctors, nurses, and pharmacists^21^^,^^56^^,^^75^^,^^77^^,^^86^^,^^94^
	Doctors, nurses, and allied health^22^^,^^35^^,^^39^^,^^43^^,^^50^^,^^53^^,^^70^^,^^107^
	Nurses only^52^^,^^59^^,^^73^
	Pharmacists only^72^^,^^85^^,^^100^^,^^102^
	Also involved senior leadership or managerial users^24^^,^^27^^,^^28^^,^^35^^,^^39^^,^^40^^,^^43^^,^^50^^,^^51^^,^^53^^,^^54^^,^^57^^,^^59^^,^^65^^,^^73^^,^^75^^,^^77^^,^^81^^,^^86^^,^^95^^,^^105^
**Audit** *What were the source(s) of data collected?*	Electronic health record data^21^^,^^24^^,^^26^^,^^28^^,^^32^^,^^35^^,^^39–41^^,^^43^^,^^53^^,^^56^^,^^57^^,^^63^^,^^65^^,^^67^^,^^69^^,^^71^^,^^72^^,^^74^^,^^75^^,^^77^^,^^79–81^^,^^84^^,^^86^^,^^91^^,^^93–95^^,^^97^^,^^98^^,^^101^^,^^104^^,^^107^
	Specific prescribing system data^27^^,^^62^^,^^65^^,^^74^^,^^89^^,^^92^^,^^100^^,^^102^
	Separate biochemistry, laboratory or radiological database^22^^,^^24^^,^^41^^,^^70^^,^^78^^,^^91^
	External national or regional database^26^^,^^37^^,^^42^^,^^48^^,^^50^^,^^54^^,^^60^^,^^73^^,^^85^^,^^99^^,^^103^^,^^105^
	Nursing data^22^^,^^41^^,^^52^^,^^59^^,^^73^^,^^75^
	Healthcare staff self-reported data^31^^,^^33^^,^^92^
	Patient reported outcomes data^25^^,^^51^^,^^103^
**Feedback display** *What element(s) were presented with the feedback?*	Graphical elements^21^^,^^22^^,^^24–28^^,^^31^^,^^33^^,^^37^^,^^40^^,^^42^^,^^43^^,^^48^^,^^50^^,^^51^^,^^53–57^^,^^59^^,^^60^^,^^67^^,^^69^^,^^72–75^^,^^77–81^^,^^84–86^^,^^89^^,^^91^^,^^93^^,^^94^^,^^96^^,^^101–103^^,^^107^
	Benchmarking^21^^,^^22^^,^^24^^,^^25^^,^^27^^,^^28^^,^^31–33^^,^^37^^,^^39^^,^^40^^,^^42^^,^^43^^,^^48^^,^^50–57^^,^^59^^,^^60^^,^^63^^,^^65^^,^^67^^,^^69^^,^^73–75^^,^^77–81^^,^^84–86^^,^^89^^,^^91–93^^,^^95–97^^,^^99–101^^,^^103–105^^,^^107^
	Patient lists^21^^,^^22^^,^^24^^,^^26^^,^^28^^,^^35^^,^^39–41^^,^^48^^,^^52^^,^^54^^,^^55^^,^^57^^,^^60^^,^^62^^,^^63^^,^^65^^,^^67^^,^^69–72^^,^^74^^,^^75^^,^^78–81^^,^^84^^,^^91^^,^^92^^,^^96–98^^,^^101–105^^,^^107^
	Detailed patient-level data^22^^,^^24^^,^^26^^,^^28^^,^^35^^,^^39^^,^^40^^,^^48^^,^^55^^,^^57^^,^^63^^,^^65^^,^^67^^,^^69^^,^^70^^,^^72^^,^^75^^,^^77–79^^,^^91^^,^^92^^,^^97^^,^^102^
	Individual Performance levels^22^^,^^25^^,^^27^^,^^31^^,^^32^^,^^35^^,^^37^^,^^40^^,^^42^^,^^43^^,^^48^^,^^50^^,^^51^^,^^53^^,^^54^^,^^56^^,^^59–61^^,^^63^^,^^65^^,^^67^^,^^69^^,^^72–74^^,^^77^^,^^79^^,^^84–86^^,^^89^^,^^91–97^^,^^99^^,^^100^^,^^103^^,^^104^^,^^107^
	Individual practice performance levels (primary care)^26^^,^^57^^,^^71^^,^^78^^,^^80^^,^^81^^,^^101^^,^^104^
	Qualitative data (free text communication)^24^^,^^52^^,^^72^^,^^91^
	Prioritization (color coding or sorting functions)^21^^,^^26^^,^^27^^,^^35^^,^^39^^,^^41^^,^^43^^,^^48^^,^^53–55^^,^^57^^,^^60^^,^^65^^,^^69^^,^^70^^,^^72^^,^^74^^,^^75^^,^^77–81^^,^^85^^,^^86^^,^^91^^,^^92^^,^^95^^,^^100–103^^,^^107^
**Co-interventions** *What other interventions were present alongside the system?*	Action plans^24^^,^^25^^,^^27^^,^^32^^,^^33^^,^^35^^,^^42^^,^^43^^,^^54–56^^,^^62^^,^^72^^,^^73^^,^^75^^,^^79^^,^^84^^,^^91^^,^^99^^,^^101^
	Financial reward or alignment^25^^,^^28^^,^^32^^,^^56^^,^^57^^,^^74^^,^^77^^,^^79^^,^^81^^,^^84^^,^^103^^,^^104^
	Clinical education^28^^,^^32^^,^^33^^,^^37^^,^^52^^,^^53^^,^^65^^,^^80^^,^^81^^,^^86^^,^^91^^,^^92^^,^^99^^,^^100^^,^^105^
	Peer discussion^25^^,^^27^^,^^37^^,^^40^^,^^43^^,^^48^^,^^59^^,^^65^^,^^80^^,^^81^^,^^86^^,^^91^^,^^103^
	External change agent^43^^,^^59^^,^^71^^,^^77^^,^^93^
	Clinical decision support, reminders, or alerts^26^^,^^32^^,^^53^^,^^57^^,^^71^^,^^72^^,^^75^^,^^79^^,^^80^^,^^84^^,^^91^^,^^95^^,^^97^^,^^102^^,^^104^
	Patient education^21^^,^^65^^,^^92^
**Organisational context** *What were the conditions and setting characteristics surrounding the system?*	Leadership support^21^^,^^24^^,^^25^^,^^27^^,^^33^^,^^35^^,^^39^^,^^40^^,^^43^^,^^50–55^^,^^57^^,^^59^^,^^65^^,^^73–75^^,^^77^^,^^80^^,^^81^^,^^85^^,^^86^^,^^93–95^^,^^100^^,^^103^^,^^105^
	Intraorganizational networks^21^^,^^24^^,^^25^^,^^27^^,^^28^^,^^33^^,^^39–41^^,^^43^^,^^50^^,^^53^^,^^55^^,^^57^^,^^59^^,^^65^^,^^70^^,^^73–75^^,^^77^^,^^78^^,^^81^^,^^86^^,^^94^^,^^100^^,^^102^^,^^103^^,^^105^
	Extraorganizational networks^37^^,^^39^^,^^40^^,^^42^^,^^43^^,^^50^^,^^53–55^^,^^57^^,^^60^^,^^65^^,^^75^^,^^79^^,^^81^^,^^85^^,^^86^^,^^99^^,^^101^^,^^103–105^
	Limited reporting of organizational support^22^^,^^26^^,^^31^^,^^48^^,^^56^^,^^61–63^^,^^67^^,^^71^^,^^72^^,^^84^^,^^89^^,^^92^^,^^96–98^^,^^101^^,^^107^
	Champions^51^^,^^55^^,^^65^^,^^74^^,^^75^^,^^77^^,^^86^^,^^105^
	Feedback delivered to a group^25^^,^^27^^,^^33^^,^^37^^,^^40^^,^^43^^,^^59^^,^^62^^,^^74^^,^^80^^,^^100^
	Workflow fit considered^21^^,^^25^^,^^28^^,^^32^^,^^55^^,^^56^^,^^69^^,^^74^^,^^80^^,^^81^^,^^84^^,^^92^^,^^103^^,^^104^
	Limited reporting of implementation process^26^^,^^33^^,^^37^^,^^40^^,^^41^^,^^50^^,^^51^^,^^56^^,^^57^^,^^61^^,^^62^^,^^67^^,^^70–72^^,^^74^^,^^78^^,^^85^^,^^92^^,^^95^^,^^96^^,^^98^^,^^99^^,^^102^^,^^104^^,^^107^

*Note:* A descriptive summary of the differing features and characteristics of e-A&F systems based on clinical performance feedback intervention theory.

### Comparison against generic A&F “best practices” (Objective 2)

Table 4 below summarizes the number of characteristics each e-A&F system had compared against a list of 18 recommended best practices for generic A&F.^1^^,^^5^ Systems adopted a median of 8 best practices (interquartile range 6–10). None of the 65 systems exhibited all 18 best practices (range 1–14). An increasing number of best practice features were adopted over time, with linear regression estimating 0.40 (95% CIs, 0.32–0.48) new features per year (Supplementary File S4).

**Table 4.** Comparison of computerized audit and feedback systems against theorized best practices

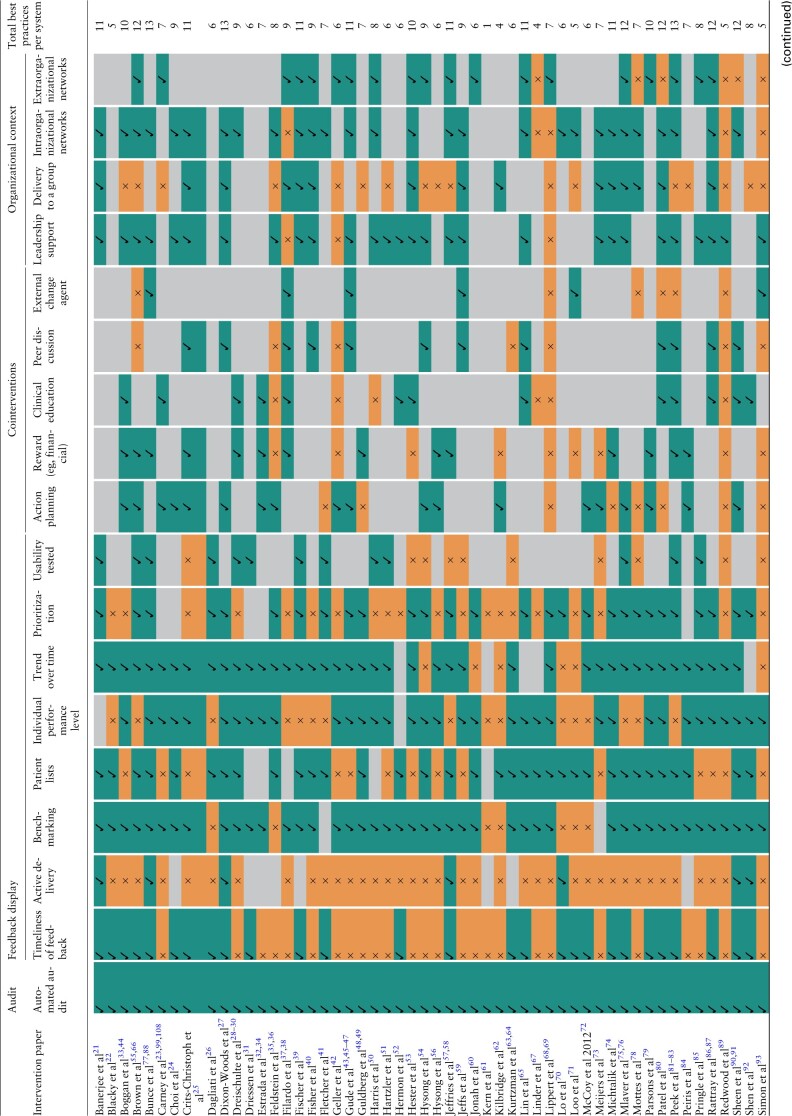 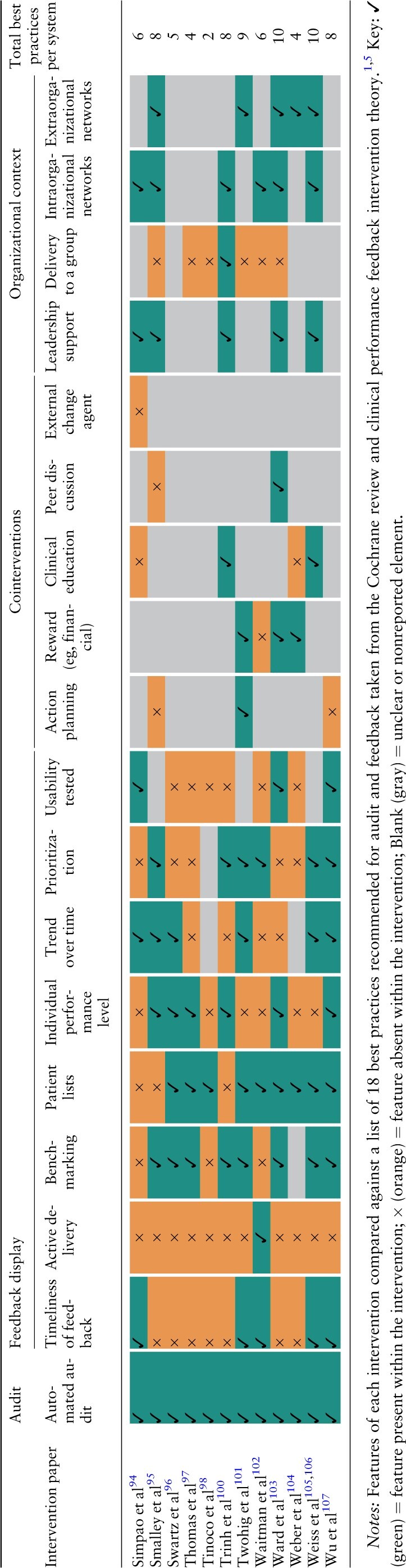

All systems adopted automated audit, with 48 systems showing data on trend over time in uses and functions. Timeliness of feedback data varied with 32 systems reporting immediate or “near real-time” feedback, and most others (21 systems) reporting feedback monthly or less frequent. “Cointerventions” that were defined as part of recommended best practices were commonly offered alongside e-A&F systems (Tables 3 and 4). Action planning was encouraged by 20 systems, with some containing embedded recommended actions within systems and others encouraging users to define their own action plans. Other common cointerventions included financial or other rewards (17 systems) and clinical education (15 systems). Organizational context was often poorly reported with 19 systems stating limited information on organizational support and 26 systems having a limited description of their implementation process. For those that specified, 33 systems had leadership support, with 34 systems involving intraorganizational networks and 24 systems involving extraorganizational networks. Intraorganization networks frequently involved management roles and included speciality committees, working groups and primary care practice teams. Extraorganizational networks were varied encompassing widespread academic networks, governmental agencies, and pharmacy chains.

### How e-A&F systems may impact patient care and outcomes (Objective 3)

**Figure 2.**
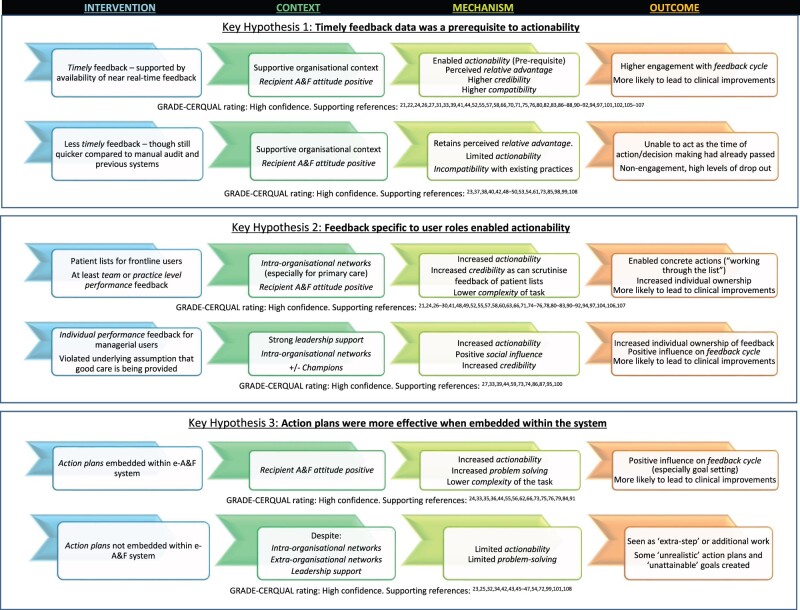
Summary of key findings on how computerized audit and feedback systems impact patient care and outcomes. It presents key findings, supported by intervention-context-mechanism-outcome (ICMO) configurations along with supporting references and GRADE-CERQual assessments.^16^^,^^19^ Three key intervention factors were identified that enhanced actionability and were more likely to result in clinical improvements, including the availability of timely data for feedback, feedback functions specific to user roles, and action plans embedded within systems. For a more comprehensive list of ICMOs see Supplementary File S5, with further descriptions and explanations of mechanism constructs in Supplementary File S6. Constructs taken from clinical performance feedback intervention theory are in italics.^5^

Key findings supported by ICMO configurations are presented in Figure 2. For readability, we focus on high confidence and novel findings related to e-A&F, with a full list of ICMO configurations and CER-QUAL ratings in Supplementary File S5. A substantial proportion of studies (over 30%) reported insignificant results or included negative findings, allowing us to compare and contrast ICMOs for these systems.^23^^,^^25^^,^^32^^,^^34^^,^^37^^,^^38^^,^^40^^,^^42^^,^^43^^,^^45–47^^,^^50^^,^^53^^,^^54^^,^^61^^,^^62^^,^^70^^,^^72^^,^^73^^,^^77^^,^^85^^,^^89^^,^^93^^,^^99^^,^^101^^,^^108^ A large majority of the codes arose from CP-FIT, though some nuanced codes building on CP-FIT were identified inductively (see Supplementary Files S5 and S6).^5^ When compared with the other mechanisms within CP-FIT, *actionability* appeared to be the most important mechanism in producing clinical improvements.^21–63^^,^^65–97^^,^^99–108^  *Actionability* is the ability of e-A&F systems to directly facilitate behaviors for users. Namely, the more an e-A&F system successfully and directly supported clinical behaviors with tangible or concrete next steps, the more users felt empowered and motivated to act on these behaviors more effectively, also increasing achievability and controllability of the task.^21–63^^,^^65–97^^,^^99–108^ Other mechanisms within CP-FIT (eg, reduced complexity, perceived relative advantage, see Supplementary File S6 for full descriptions and explanations) often contributed to successful e-A&F systems, but were less important as influencing factors, and were insufficient to produce clinical improvements alone.^23^^,^^25^^,^^32^^,^^34^^,^^37^^,^^38^^,^^40^^,^^42^^,^^43^^,^^45–47^^,^^50^^,^^53^^,^^54^^,^^61^^,^^62^^,^^70^^,^^72^^,^^73^^,^^77^^,^^85^^,^^89^^,^^93^^,^^99^^,^^101^^,^^108^^,^^109^ Contextual factors were also key effect modifiers of e-A&F systems, as they significantly enabled or limited implementation and engagement with each system.^21^^,^^23–25^^,^^27–30^^,^^32–35^^,^^37–59^^,^^63^^,^^65–70^^,^^73–91^^,^^93–95^^,^^99–108^ However, despite strong organization and contextual backing, systems without actionable feedback were unlikely to result in clinical improvements.^23^^,^^25^^,^^32^^,^^34^^,^^37^^,^^38^^,^^40^^,^^42^^,^^43^^,^^45–47^^,^^50^^,^^53^^,^^54^^,^^61^^,^^62^^,^^70^^,^^72^^,^^73^^,^^77^^,^^85^^,^^89^^,^^93^^,^^99^^,^^101^^,^^108^^,^^109^

Three key e-A&F intervention factors were identified that enhanced actionability and were more likely to result in clinical improvements:

- The availability of real-time data for feedback
- Feedback functions specific to user roles
- Action plans embedded within systems

#### Timely feedback data as a prerequisite to actionability

Systems that provided immediately updated or “near real-time” feedback resulted in higher engagement and were more likely to report successful outcomes.^21^^,^^22^^,^^24^^,^^26^^,^^27^^,^^31^^,^^33^^,^^39^^,^^41^^,^^44^^,^^52^^,^^55^^,^^57^^,^^66^^,^^70^^,^^71^^,^^74–77^^,^^79–83^^,^^86–88^^,^^90–92^^,^^94^^,^^97^^,^^102^^,^^105–107^ The timeliness of feedback enabled the data to be viewed as more credible and representative of performance.^21^^,^^22^^,^^24^^,^^26^^,^^27^^,^^31^^,^^33^^,^^39^^,^^41^^,^^44^^,^^52^^,^^55^^,^^57^^,^^58^^,^^66^^,^^70^^,^^71^^,^^75^^,^^76^^,^^80^^,^^82^^,^^83^^,^^86–88^^,^^90–92^^,^^94^^,^^97^^,^^101^^,^^102^^,^^105–107^ Importantly, it was reported as a prerequisite for actionability, with less timely feedback frequently been seen as extra work, and occurring outside existing workflow.^24^^,^^27^^,^^55^^,^^77^^,^^83^^,^^102^^,^^107^ Although almost all systems provided more timely feedback compared with manual audit and previous systems, several of these studies reported that without immediate feedback, it remained too long for effective action to be taken despite many users finding the feedback “helpful” or “insightful.” ^40^^,^^48–50^^,^^53^^,^^54^^,^^61^^,^^73^^,^^85^^,^^98^ Likewise, the lack of real-time feedback was reported to be a barrier to usage in several studies.^48–50^^,^^53^^,^^73^^,^^85^

No e-A&F systems providing annual feedback reported significant improvements in patient care, with several studies reporting low usage and high dropout.^23^^,^^37^^,^^38^^,^^42^^,^^50^^,^^61^^,^^73^^,^^99^^,^^108^ For instance, the “Web-based Tailored Educational Intervention Data System” only produced yearly feedback for users once, with only 55% of enrolled participants using the system and a large dropout and null effect by the end.^99^ This was despite more than 80% of users rating the intervention “very helpful” in several domains including that the feedback was useful to evaluate their practice.^23^ Similarly, a web-based benchmarking tool for heart failure and pneumonia provided annual retrospective data and received >50% dropout rate by the end of the study, failing to detect any differences in care performance.^37^^,^^38^

#### Feedback specific to user roles enabled actionability

e-A&F systems were designed for a wide range of users that fell into 2 main roles. The majority were “frontline” users responsible for delivering care (eg, doctors, nurses, pharmacists), with others being “managerial” users (eg, managers, leadership, or organizational roles). To be directly actionable, feedback needed to be specific to user roles: feedback to “frontline” users mainly required patient lists, whereas for feedback to “managerial” users, the priority was highlighting the specifics for individual performance. Many successful systems presented specific feedback on both patient lists and individual or practice performance levels,^24^^,^^27–30^^,^^35^^,^^36^^,^^48^^,^^60^^,^^68^^,^^69^^,^^74^^,^^77–80^^,^^84^^,^^88^^,^^90^^,^^91^^,^^96^^,^^97^^,^^103^^,^^107^ with various using functions such as color coding and sorting , ^21^^,^^26^^,^^27^^,^^35^^,^^36^^,^^41^^,^^48^^,^^49^^,^^55^^,^^57^^,^^58^^,^^60^^,^^66^^,^^68–70^^,^^74–80^^,^^83^^,^^90–92^^,^^102^^,^^107^ to enhance prioritization of actions to be taken.

Patient lists to “frontline” users generally highlighted gaps in recommended care, supported by team or practice level performance feedback (particularly for primary care).^26^^,^^55^^,^^57^^,^^58^^,^^66^^,^^71^^,^^81–83^^,^^104^ These electronic patient lists, were seen as more efficient than standard care, with the e-A&F system reporting superior effects to alerts within the electronic medical record.^57^^,^^71^^,^^80^^,^^104^ Many studies without user-specific feedback including lack of patient lists,^23^^,^^37^^,^^38^^,^^42^^,^^53^^,^^85^^,^^89^^,^^93^^,^^99^^,^^108^ or individual performance data,^40^^,^^61^^,^^62^^,^^70^ did not demonstrate significant improvements to patient outcomes. Several of these studies reported specificity of the data (both on an individual practitioner level and a patient level) to be a barrier to actionability and usage.^37^^,^^38^^,^^54^^,^^85^ For example, Filardo et al^37^^,^^38^^,^^109^ described a benchmarking and case review tool, which combined education initiatives with feedback on aggregate measures, rather than highlighting individual performances.^37^^,^^38^^,^^109^ This resulted in no significant effects on patient care, with only 26% completing the full intervention.^37^^,^^38^

Nevertheless, within a strong organizational context, individual clinician performance feedback (even without patient lists) given to “managerial” users or senior staff, particularly from leadership or management, was also effective.^27^^,^^33^^,^^39^^,^^44^^,^^59^^,^^73^^,^^74^^,^^86^^,^^87^^,^^95^^,^^100^ Although this entailed an extra step to deliver feedback to frontline care staff and often required good interdisciplinary collaboration, the process appeared to increase motivation and accountability.^27^^,^^39^^,^^59^^,^^86^^,^^87^^,^^95^^,^^100^ This process influenced individual users to take ownership of the feedback, including the responsibility to directly address the care gaps highlighted and prevented the assumption that someone else would.^27^^,^^39^^,^^54^^,^^73^^,^^74^^,^^86^^,^^87^^,^^95^^,^^100^ For example, Dixon-Woods et al^27^ described how the leadership team closely scrutinized the data and set up meetings that effectively targeted individuals who were underperforming in one area or another. With a strong “improvement culture” led by the leadership team, staff viewed their own feedback critically and over time, enabled downstream improvements even without prompts from the leadership team.^27^ In contrast, Crits-Christoph et al^25^ designed a system to collect performance ratings of therapeutic alliance, treatment satisfaction, and drug and alcohol use. To protect clinician employment and confidentiality, individual clinicians and patients could not be identified and so users struggled to act on the feedback.^25^ Despite monthly meetings, leadership support, and financial incentives, no significant improvements in clinical outcomes measures were noted.^25^

#### Action plans were more effective when embedded within the system

The e-A&F systems that incorporated action plans as part of their multi-faceted interventions appeared to produce better results.^24^^,^^33^^,^^35^^,^^36^^,^^44^^,^^55^^,^^56^^,^^62^^,^^66^^,^^73^^,^^75^^,^^76^^,^^79^^,^^84^^,^^91^ For example, Feldstein et al^35^^,^^36^ designed a dashboard that showed not only color-coded graphs of clinical performance compared with guidelines but also had a list of prompts for how to achieve recommended targets for individual patients (eg, prompts to conduct a screening test or adjusting a medication dose). This resulted in significant improvements in care scores for several chronic disease areas, with users feeling “empowered” to proactively manage wider patient needs, particularly for broader clinical roles.^35^ Similarly, a website reported percentages of patients meeting BP targets primary care professionals, and importantly included suggested actions designed to be simple and achievable.^56^ This allowed direct actions to address gaps in performance and resulted in significant increases in the use of guideline-recommended medications for blood pressure.^56^

Conversely, when users were asked to come up with their own action plan either as part of meetings or as part of wider quality improvement activity groups, it reduced actionability, and at times resulted in unrealistic action plans and unattainable goals.^23^^,^^25^^,^^32^^,^^34^^,^^42^^,^^43^^,^^45–47^^,^^54^^,^^72^^,^^99^^,^^101^^,^^108^ In a medication safety system targeting patients with acute kidney injury, pharmacists input their own recommendations for doctors, rather than doctors being able to direct action changes in medication.^72^ This resulted in a time delay before the action plan could be implemented and no improvements in adverse drug reactions or time taken to stop nephrotoxic medications.^72^

## DISCUSSION

This review summarized 88 studies of e-A&F systems, demonstrating their wide range of settings, applications, and characteristics. Despite automated audit and advantages in analysis compared with manual methods, it was insufficient for e-A&F systems to just feedback more data, or solely present measurements and targets for performance. When compared with generic A&F best practices, there was an increased expectation for e-A&F systems to present more precise and nuanced feedback, to make it easy to act on or present viable next steps to improve patient care. Established effective components of wider A&F interventions include timely feedback, individualized feedback and action planning.^1^^,^^5^ Yet, even some recent e-A&F systems lacked these, with extensive inconsistencies between different systems. Our review highlights more nuanced requirements for e-A&F, including the availability of immediate or ‘near real-time’ data for feedback; feedback functions that were specific to user roles (including “patient lists” for frontline users and “individual performance feedback” for senior or managerial users); and embedding action plans within systems. A key consideration for successful e-A&F was enabling feedback to be actionable, yet underlying contexts of organizations, resources, and user characteristics deeply affected the uptake of e-A&F systems, considerably influencing their effects in several studies.

### Comparison with existing literature

Our review builds on wider evidence regarding A&F, revealing important findings for computerized interventions.^1^^,^^3^^,^^4^ In particular, e-A&F systems offer opportunities to enhance the positive effects of 3 known generic A&F best practices, including timeliness, specificity, and action-planning.^1^^,^^3^^,^^4^^,^^6^^,^^10^ Our findings present a more explicit understanding of these, recommending the provision of real-time data, feedback functions tailored to user roles (particularly patient lists to frontline users and individual performance data to managerial roles), along with embedded action plans. With an increasing uptake of e-A&F, wider A&F best practices could be extended to take these into account.^1^^,^^5^^,^^6^ Our review utilized a list of 18 best practices, focusing on more objective features to aid clarity, but this was only one way of classifying e-A&F system components. Though there is considerable overlap, others have proposed slightly different classifications.^4^^,^^6^^,^^10^^,^^110^ Our approach was guided by the reporting within papers, and explicitly considered organizational factors and cointerventions, though omitted more complex and subjective characteristics that were less evidently reported, such as trust or identity.^1^^,^^5^^,^^6^^,^^10^

Two systematic reviews on e-A&F systems have been performed previously in 2015 and 2017. Dowding et al (2015) included 11 studies on dashboards, highlighting that contextual factors were key to the usage of e-A&F systems and hence the effect on outcomes. Tuti et al^7^ examined 7 RCTs, but noted highly heterogeneous effect sizes. Our review builds on these findings, adopting broader inclusion criteria to examine a wider range of studies in a narrative synthesis to identify characteristics of e-A&F systems more likely to result in care improvements. Consistent with findings from these 2 previous reviews, several contextual factors within included “best practices” appeared to be beneficial in encouraging the uptake of systems and positive outcomes. In particular, leadership support and intraorganizational networks appeared to support user role-specific feedback, strengthening motivation and accountability to act on feedback data.

### Implications for practice

This review compliments wider literature in advocating an “action over measurement” approach.^111^^,^^112^ With limited time and resources in healthcare, actionability within e-A&F systems appears important to enable tangible changes in care, rather than simply chasing targets or measuring performance.^113^ Important features highlighted by this review to enable actionability include the availability of real-time data, feedback specific to user roles, and embedded action plans. However, even some recent systems lacked basic features recommended by best practice, such as timely feedback and action planning. With e-A&F systems increasing in their potential functions and complexity, it suggests a need for codesign with relevant stakeholders to increase usability, participation, and sustainability that takes into account theorized “best practices.”^114^^,^^115^ Otherwise, with increasing complexity, computerized tools are more likely to result in nonadoption and abandonment.^116^^,^^117^ Enhancing functionality of e-A&F systems alone would be futile if computerized tools failed in their uptake, implementation, or sustainability.

### Strengths and limitations

This is the largest review of studies focusing on e-A&F to date. It incorporated CP-FIT and applied realist principles in exploring a wide range of literature, from RCTs to qualitative studies to generate a rich insight into the current state of e-A&F systems. Our synthesis considered all studies regardless of methodological quality but was guided by our quality appraisal and GRADE-CERQual assessment in the confidence of findings. Applying CP-FIT allowed a greater depth of analysis based on theoretical findings for wider A&F and a framework of hypothesized “best practices.” However, use of CP-FIT may at the same time have limited novel themes, as findings may have been biased to preformed constructs. Through CP-FIT, we aimed to extend existing knowledge frameworks on wider A&F through application to e-A&F systems. Though we attempted to focus on findings specific to e-A&F, it was not always possible to ascertain whether features for success or failure were specific to just e-A&F or inherent to A&F interventions more generally.

As with other literature syntheses, our results are limited to the reporting and transparency of the authors within original studies. Though we propose and prioritize key mechanisms for success, our review was not designed to quantify casual effects or relative effect sizes. There is a degree of uncertainty in our highlighted mechanisms having a significant casual effect on process and outcomes and it is possible that underreported features may have greater effects on patient care. Our review likely identified studies with a predisposition towards recruiting participants from organizations with better resources and infrastructures, particularly in information technology, and hence our findings may be less applicable to low resource settings. We also restricted our search to published articles within medical databases and Google scholar to focus on systems for healthcare, but searching of further technology focused databases (eg, IEEE Xplore and ACM Digital Library) may have yielded further studies. Iterative interpretation of data is a core component of realist synthesis, but this has obvious implications for the replication of findings from the review, as others may have interpreted the evidence differently.

## CONCLUSIONS

e-A&F systems continue to be developed for a wide range of clinical applications. Yet, it remains that several systems still lack basic features recommended by best practice, such as timely feedback and action planning. e-A&F systems should consistently incorporate best practices that enhance actionability by using real-time data, feeding back in ways that are specific to user roles, and providing embedded action plans. Future research needs to address inconsistencies in e-A&F system features, to ensure development incorporates features recommended by best practice, which can increase actionability of feedback and may improve outcomes.

## FUNDING

This article is linked to independent research funded by the National Institute for Health Research (NIHR) through the Greater Manchester Patient Safety Translational Research Centre (award No. PSTRC-2016-003).

## AUTHOR CONTRIBUTIONS

JT and BB conceived of the article and developed the study design. JT and BB performed study selection, screening, extraction, and quality appraisal. Results were developed by JT and BB under the supervision of SV and NP. JT wrote the article with contributions and comments from BB, SV, NP, IB. JT is guarantor of the article.

## SUPPLEMENTARY MATERIAL

Supplementary material is available at *Journal of the American Medical Informatics Association* online.

## CONFLICT OF INTEREST STATEMENT

The authors declare that they have no competing interests. The views expressed in this document are those of the authors and not necessarily those of the NHS, NIHR, or the Department of Health and Social Care.

## DATA AVAILABILITY

The data supporting the findings of this study are available within Supplementary Files, with further datasets available upon reasonable request.

## Supplementary Material

ocac031_Supplementary_Data
