# Supplementary material for: Systematic review and narrative synthesis of computerized audit and feedback systems in healthcare
Source: J Am Med Inform Assoc. 2022 Mar 10;29(6):1106–19. doi: 10.1093/jamia/ocac031 (PMC9093027; doi:10.1093/jamia/ocac031)
Supplement: ocac031_Supplementary_Data [file ocac031_supplementary_data.zip › Additional File 1 (search terms).docx]

## Additional file 1: Search terms and results

1. **MEDLINE (Ovid) 1946 to February Week 2 2021 – searched 18th Feb 2021**

| 1 | (audit* adj3 feedback).tw. | 2914 |
| --- | --- | --- |
| 2 | Clinical Audit/ | 1896 |
| 3 | Medical Audit/ | 17269 |
| 4 | Nursing Audit/ | 3012 |
| 5 | Dental Audit/ | 424 |
| 6 | Management Audit/ | 2525 |
| 7 | Benchmarking/ | 14369 |
| 8 | "Commission on Professional and Hospital Activities"/ | 288 |
| 9 | Feedback/ | 30716 |
| 10 | Feedback, Psychological/ | 3627 |
| 11 | Utilization Review/ | 8126 |
| 12 | Drug Utilization Review/ | 3831 |
| 13 | Concurrent Review/ | 383 |
| 14 | Peer Review, Health Care/ | 1446 |
| 15 | (audit or audits or auditing).tw. | 36308 |
| 16 | feedback.tw. | 118826 |
| 17 | (review adj3 record?).tw. | 15495 |
| 18 | chart review.tw. | 35878 |
| 19 | (practice data or hospital* data).tw. | 5902 |
| 20 | benchmark*.tw. | 31543 |
| 21 | or/2-20 | 283117 |
| 22 | exp Health Personnel/ | 545868 |
| 23 | exp Hospitals/ | 287873 |
| 24 | exp Professional Practice/ | 257574 |
| 25 | Family Practice/ | 65677 |
| 26 | Professional Competence/ | 24740 |
| 27 | Clinical Competence/ | 97849 |
| 28 | Physician's Practice Patterns/ | 63333 |
| 29 | Nurse's Practice Patterns/ | 2885 |
| 30 | Dentist's Practice Patterns/ | 2431 |
| 31 | Quality Assurance, Health Care/ | 56504 |
| 32 | Quality of Health Care/ | 75002 |
| 33 | ((health* personnel or health care personnel or physician? or doctor? or clinician? or nurse? or provider? or practitioner? or resident? or professional? or nursing or clinical) adj3 (skill or skills or behaviour or behavior or competence)).tw. | 41762 |
| 34 | ((clinical or medical or dental or private or general or family or professional or hospital?) adj practice?).tw. | 259235 |
| 35 | (practice pattern? or pattern of practice).tw. | 7865 |
| 36 | (quality adj (assurance or improvement or control)).tw. | 93401 |
| 37 | (healthcare quality orhealthcare quality orquality ofhealthcare or quality of health care or quality of care).tw. | 50392 |
| 38 | performance.tw. | 794463 |
| 39 | ((influenc* or chang*) adj3 (behaviour* or behavior*)).tw. | 76843 |
| 40 | or/22-39 | 2322010 |
| 41 | 21 and 40 | 88852 |
| 42 | 1 or 41 | 90363 |
| 43 | Animals/ | 6865062 |
| 44 | Humans/ | 19461663 |
| 45 | 43 not (43 and 44) | 4821499 |
| 46 | 42 not 45 | 88730 |
| 47 | (Electronic adj3 (feedback or medical record? or health record?)).tw. | 29174 |
| 48 | ((application or design or monitoring or prototype) adj3 software).tw. | 3653 |
| 49 | (Web adj3 (based or report or worldwide)).tw. | 28522 |
| 50 | Website.tw. | 15103 |
| 51 | Internet based.tw. | 7504 |
| 52 | Visual analytics.tw. | 267 |
| 53 | Touchscreen.tw. | 723 |
| 54 | Panel-management.tw. | 50 |
| 55 | Information display.tw. | 101 |
| 56 | user interface.tw. | 4933 |
| 57 | Health informatic?.tw. | 1584 |
| 58 | health information technology.tw. | 2368 |
| 59 | Clinical decision support system?.tw. | 1763 |
| 60 | ((Computer? or Computeri?ed) adj3 (strategy or strategies or software? or tool? or system? or technology)).tw. | 29996 |
| 61 | or/47-60 | 118413 |
| 62 | 46 and 61 | 4198 |
| 63 | ((Clinical or system? or digital or electronic or performance or quality or surveillance or visual or visuali?ation) adj3 dashboard).tw. | 248 |
| 64 | 62 or 63 | 4421 |
| 65 | ..l/ 64 yr=1999-2020 | 4060 |
| 66 | limit 65 to (abstracts and english language) | 3956 |
| 67 | limit 65 to (addresses or autobiography or clinical conference or editorial or interview or letter or news or newspaper article or "review" or systematic reviews) | 289 |
| 68 | 66 not 67 | 3687 |
| **69** | **remove duplicates from 68** | **3686** |

1. **EMBASE(Ovid) 1980 to 2021 Week 6 – searched 18th Feb 2021**

| 1 | (audit* adj3 feedback).tw. | 4409 |
| --- | --- | --- |
| 2 | Clinical Audit/ | 5904 |
| 3 | Medical Audit/ | 48843 |
| 4 | Nursing Audit/ | 37 |
| 5 | Dental Audit/ | 5904 |
| 6 | Management Audit/ | 47701 |
| 7 | Benchmarking/ | 6669 |
| 8 | "Commission on Professional and Hospital Activities"/ | 5904 |
| 9 | Feedback/ | 61818 |
| 10 | Feedback, Psychological/ | 404 |
| 11 | Utilization Review/ | 55350 |
| 12 | Drug Utilization Review/ | 675 |
| 13 | Concurrent Review/ | 55350 |
| 14 | Peer Review, Health Care/ | 32394 |
| 15 | (audit or audits or auditing).tw. | 87619 |
| 16 | feedback.tw. | 186224 |
| 17 | (review adj3 record?).tw. | 28931 |
| 18 | chart review.tw. | 90039 |
| 19 | (practice data or hospital* data).tw. | 11625 |
| 20 | benchmark*.tw. | 56826 |
| 21 | or/2-20 | 609598 |
| 22 | exp Health Personnel/ | 1646913 |
| 23 | exp Hospitals/ | 1181148 |
| 24 | exp Professional Practice/ | 365380 |
| 25 | Family Practice/ | 71872 |
| 26 | Professional Competence/ | 32533 |
| 27 | Clinical Competence/ | 61958 |
| 28 | Physician's Practice Patterns/ | 256816 |
| 29 | Nurse's Practice Patterns/ | 4666 |
| 30 | Dentist's Practice Patterns/ | 290611 |
| 31 | Quality Assurance, Health Care/ | 189984 |
| 32 | Quality of Health Care/ | 202532 |
| 33 | ((health* personnel or health care personnel or physician? or doctor? or clinician? or nurse? or provider? or practitioner? or resident? or professional? or nursing or clinical) adj3 (skill or skills or behaviour or behavior or competence)).tw. | 61994 |
| 34 | ((clinical or medical or dental or private or general or family or professional or hospital?) adj practice?).tw. | 406328 |
| 35 | (practice pattern? or pattern of practice).tw. | 14433 |
| 36 | (quality adj (assurance or improvement or control)).tw. | 165321 |
| 37 | (healthcare quality orhealthcare quality orquality ofhealthcare or quality of health care or quality of care).tw. | 78168 |
| 38 | performance.tw. | 1246517 |
| 39 | ((influenc* or chang*) adj3 (behaviour* or behavior*)).tw. | 111511 |
| 40 | or/22-39 | 4601190 |
| 41 | 21 and 40 | 251407 |
| 42 | 1 or 41 | 253114 |
| 43 | Animals/ | 1174584 |
| 44 | Humans/ | 15604705 |
| 45 | 43 not (43 and 44) | 888852 |
| 46 | 42 not 45 | 252634 |
| 47 | ((application or design or monitoring or prototype) adj3 software).tw. | 7058 |
| 48 | Internet based.tw. | 12297 |
| 49 | Visual analytics.tw. | 540 |
| 50 | Touchscreen.tw. | 1355 |
| 51 | Panel-management.tw. | 158 |
| 52 | Information display.tw. | 145 |
| 53 | user interface.tw. | 7656 |
| 54 | Health informatic?.tw. | 2126 |
| 55 | health information technology.tw. | 3066 |
| 56 | Clinical decision support system?.tw. | 2693 |
| 57 | ((Computer? or Computeri?ed) adj3 (strategy or strategies or software? or tool? or system? or technology)).tw. | 43070 |
| 58 | ((Clinical or system? or digital or electronic or performance or quality or surveillance or visual or visuali?ation) adj3 dashboard).tw. | 608 |
| 59 | or/47-58 | 78637 |
| 60 | 46 and 59 | 3569 |
| 61 | ..l/ 60 yr=1999-2020 | 3096 |
| 62 | limit 61 to (abstracts and english language) | 3009 |
| 63 | limit 61 to medline | 765 |
| 64 | limit 61 to (conference abstract or editorial or erratum or letter or note or "review") | 1452 |
| **65** | **62 not (63 or 64)** | **886** |

1. **CINAHL Plus (Ebsco) 1937 to present – searched 18th Feb 2021**

| **S55** | **s54 - Limiters - Exclude MEDLINE records** | **12** |
| --- | --- | --- |
| S54 | S39 AND S53 | 2,761 |
| S53 | S40 OR S41 OR S42 OR S43 OR S45 OR S46 OR S47 OR S48 OR S49 OR S50 OR S51 OR S52 | 70,697 |
| S52 | TI Clinical decision support system? OR AB Clinical decision support system? | 1,200 |
| S51 | TI health information technology OR AB health information technology | 3,951 |
| S50 | TI health informatics OR AB health informatics | 3,330 |
| S49 | TI user W0 interface OR AB user W0 interface | 1,147 |
| S48 | TI Panel W0 management OR AB Panel W0 management | 40 |
| S47 | TI Touchscreen OR AB Touchscreen | 266 |
| S46 | TI Internet based OR AB Internet based | 4,969 |
| S45 | TI website OR AB website | 16,942 |
| S44 | TI website OR AB website | 16,942 |
| S43 | TI ( (Web) W3 (based or report) ) OR AB ( (Web) W3 (based or report) ) | 14,703 |
| S42 | TI ( ((Electronic) W3 (feedback or medical record? or health record?)). ) OR AB ( ((Electronic) W3 (feedback or medical record? or health record?)) ) | 19,611 |
| S41 | TI Dashboard? OR AB Dashboard? | 787 |
| S40 | TI ( ((Computer? or Computeri?ed) W3 (strateg* or software? or tool? or system? Or technology)) ) OR AB ( ((Computer? or Computeri?ed) W3(strateg* or software? or tool? or system? Or technology)) ) | 9,174 |
| S39 | S37 or S38 | 47,791 |
| S38 | S13 and S36 | 46,955 |
| S37 | TI ( audit* and feedback ) or AB ( audit* and feedback ) | 2,134 |
| S36 | S14 or S15 or S16 or S17 or S18 or S19 or S20 or S21 or S22 or S23 or S24 or S25 or S26 or S27 or S28 or S29 or S30 or S31 or S32 or S33 or S34 or S35 | 1,294,205 |
| S35 | TI ( influenc* N3 behaviour* or influenc* N3 behavior* or chang* N3 behaviour* or chang* N3 behavior* ) or AB ( influenc* N3 behaviour* or influenc* N3 behavior* or chang* N3 behaviour* or chang* N3 behavior* ) | 31,520 |
| S34 | TI performance or AB performance | 185,925 |
| S33 | TI ( “health care quality” or “healthcare quality” or quality W1 healthcare or quality W2 care ) or AB ( “health care quality” or “healthcare quality” or quality W1 healthcare or quality W2 care ) | 46,176 |
| S32 | TI ( quality W0 assurance or quality W0 improvement or quality W0 control ) or AB ( quality W0 assurance or quality W0 improvement or quality W0 control ) | 31,711 |
| S31 | TI practice N1 pattern* or AB practice N1 pattern* | 3,797 |
| S30 | TI ( clinical W0 practice* or medical W0 practice* or dental W0 practice* or private W0 practice* or general W0 practice* or family W0 practice* or professional W0 practice* or hospital* W0 practice* ) or AB ( clinical W0 practice* or medical W0 practice* or dental W0 practice* or private W0 practice* or general W0 practice* or family W0 practice* or professional W0 practice* or hospital* W0 practice* ) | 111,839 |
| S29 | TI ( “health personnel” N3 competence or “healthcare personnel” N3 competence or “health care personnel” N3 competence or physician N3 competence or physicians N3 competence or doctor N3 competence or doctors N3 competence or clinician N3 competence or clinicians N3 competence or nurse N3 competence or nurses N3 competence or provider N3 competence or providers N3 competence or practitioner N3 competence or practitioners N3 competence or resident N3 competence or residents N3 competence or profe [...](javascript:showHistoryTerm('ctl00_ctl00_FindField_FindField_historyControl_HistoryRepeater_ctl26_ellipsis',true)) | 2,762 |
| S28 | TI ( “health personnel” N3 behavior or “healthcare personnel” N3 behavior or “health care personnel” N3 behavior or physician N3 behavior or physicians N3 behavior or doctor N3 behavior or doctors N3 behavior or clinician N3 behavior or clinicians N3 behavior or nurse N3 behavior or nurses N3 behavior or provider N3 behavior or providers N3 behavior or practitioner N3 behavior or practitioners N3 behavior or resident N3 behavior or residents N3 behavior or professional N3 behavior or professiona [...](javascript:showHistoryTerm('ctl00_ctl00_FindField_FindField_historyControl_HistoryRepeater_ctl27_ellipsis',true)) | 9,047 |
| S27 | TI ( “health personnel” N3 behaviour or “healthcare personnel” N3 behaviour or “health care personnel” N3 behaviour or physician N3 behaviour or physicians N3 behaviour or doctor N3 behaviour or doctors N3 behaviour or clinician N3 behaviour or clinicians N3 behaviour or nurse N3 behaviour or nurses N3 behaviour or provider N3 behaviour or providers N3 behaviour or practitioner N3 behaviour or practitioners N3 behaviour or resident N3 behaviour or residents N3 behaviour or professional N3 behavi [...](javascript:showHistoryTerm('ctl00_ctl00_FindField_FindField_historyControl_HistoryRepeater_ctl28_ellipsis',true)) | 7,340 |
| S26 | TI ( “health personnel” N3 skills or “healthcare personnel” N3 skills or “health care personnel” N3 skills or physician N3 skills or physicians N3 skills or doctor N3 skills or doctors N3 skills or clinician N3 skills or clinicians N3 skills or nurse N3 skills or nurses N3 skills or provider N3 skills or providers N3 skills or practitioner N3 skills or practitioners N3 skills or resident N3 skills or residents N3 skills or professional N3 skills or professionals N3 skills or nursing N3 skills or [...](javascript:showHistoryTerm('ctl00_ctl00_FindField_FindField_historyControl_HistoryRepeater_ctl29_ellipsis',true)) | 48,829 |
| S25 | TI ( “health personnel” N3 skill or “healthcare personnel” N3 skill or “health care personnel” N3 skill or physician N3 skill or physicians N3 skill or doctor N3 skill or doctors N3 skill or clinician N3 skill or clinicians N3 skill or nurse N3 skill or nurses N3 skill or provider N3 skill or providers N3 skill or practitioner N3 skill or practitioners N3 skill or resident N3 skill or residents N3 skill or professional N3 skill or professionals N3 skill or nursing N3 skill or clinical N3 skill ) [...](javascript:showHistoryTerm('ctl00_ctl00_FindField_FindField_historyControl_HistoryRepeater_ctl30_ellipsis',true)) | 4,624 |
| S24 | (MH “Quality of Nursing Care”) | 76,253 |
| S23 | (MH “Quality of Health Care”) | 121,108 |
| S22 | (MH “Quality Assurance”) | 14,405 |
| S21 | (MH “Prescribing Patterns”) | 21,077 |
| S20 | (MH “Practice Patterns”) | 7,890 |
| S19 | (MH “Nursing Skills”) | 12,714 |
| S18 | (MH “Clinical Competence”) | 6,279 |
| S17 | (MH “Professional Competence”) | 42,583 |
| S16 | (MH “Professional Practice+”) | 17,824 |
| S15 | (MH “Hospitals+”) | 304,718 |
| S14 | (MH “Health Personnel+”) | 588,652 |
| S13 | S1 or S2 or S3 or S4 or S5 or S6 or S7 or S8 or S9 or S10 or S11 or S12 | 107,350 |
| S12 | TI benchmark* or AB benchmark* | 9,299 |
| S11 | TI hospital* W0 data or AB hospital* W0 data | 2,444 |
| S10 | TI “practice data” or AB “practice data” | 773 |
| S9 | TI “chart review” or AB “chart review” | 13,245 |
| S8 | TI review N3 record* or AB review N3 record* | 8,116 |
| S7 | TI feedback or AB feedback | 32,705 |
| S6 | TI ( audit or audits or auditing or feedback ) or AB ( audit or audits or auditing or feedback ) | 53,424 |
| S5 | (MH “Utilization Review”) | 2,164 |
| S4 | (MH “Feedback”) | 15,443 |
| S3 | (MH “Benchmarking”) | 7,435 |
| S2 | (MH “Nursing Audit”) | 936 |
| S1 | (MH “Audit”) | 17,859 |
