# Supplementary material for: Systematic review and narrative synthesis of computerized audit and feedback systems in healthcare
Source: J Am Med Inform Assoc. 2022 Mar 10;29(6):1106–19. doi: 10.1093/jamia/ocac031 (PMC9093027; doi:10.1093/jamia/ocac031)
Supplement: ocac031_Supplementary_Data [file ocac031_supplementary_data.zip › Additional File 2 (Data extraction form).docx]

# Additional File 2: Data extraction form

| **Study ID and citation** |
| --- |
|  |
| **Country of study** |
|  |
| **Setting of study**  E.g. primary care, secondary care |
|  |
| **Focus / aim of software**  E.g. to reduce medication errors / improve blood pressure control |
|  |
| **Aim statement**   - What were the stated aims of the study? (Copy and paste if possible) - What was the research question? |
|  |
| **Study design**   - What was the design of the study reported in the paper?   - Typical usability studies: heuristic evaluation, cognitive walkthrough, end user testing, focus groups, surveys   - Typical effectiveness studies: RCT, before / after study, cohort studies - Who were the study participants? |
|  |
| **Study methods**   - What were the methods of the study? |
|  |
| **Study findings:**   - What were the main findings of the study? (These can be copied and pasted) - We are particularly interested in:   - Generalisable usability findings for eA&F systems   - Effectiveness of the software related to its aims described above |
|  |
| **Use of theory in evaluation (both usability and / or effectiveness)**  Were any theoretical models used to inform the design or analysis of the evaluations? |
|  |

**SOFTWARE DETAILS**

| **Intended users of software**  Who is intended to use the software? |
| --- |
|  |
| **How was the study/software implemented?**  How did they authors invite participants into the study and how many times? |
|  |
| **Proposed mechanism of the software**   - How will the users use the software? - How is it intended that the software will achieve the aims mentioned above? - What will they do with the information in the software? - *Highlight whether this is explained by authors or if inferred by reviewer* |
|  |
| **Platform for software**   - How is the software presented - E.g. web-based, integrated into EHR |
|  |
| **Software interface design features**   - What are the main features of the software interface? - E.g. use of tables, graphics, interactive components  1. Summary of clinical performance 2. Patient lists 3. Patient-level data 4. Recommended actions |
|  |
| **Relative advantage**   - Authors’ claims for how and in what circumstances their software outperforms others - Your claims |
|  |
| **Concerns**   - Authors’ stated concerns about their software - Your concerns |
|  |
| **Audit**   - What data was collected from the software |
|  |
| **Quality indicators used in software**   - Give an example / list all of quality indicators used - Where are they taken from? (E.g. clinical guidelines, local consensus process) |
|  |
| **Quality indicator algorithms used**   - How are the quality indicators calculated? - E.g. SQL, machine-learning |
|  |
| **Quality indicator algorithm development**   - How were they developed? - Were they based on existing knowledge / literature? - Were they tested? |
|  |
| **Quality indicator algorithm accuracy**   - Have they been validated? - How accurate are they? |
|  |
| **Interface design informing evidence**   - Were design guidelines used? - Were design features based on existing evidence? - Was any theoretical models used to inform the design e.g. behaviour change theory? - Was it based / developed based on end-user needs assessment / testing? |
|  |

**QUALITY APPRAISAL**

| **1. Qualitative** | Y/N and comments |
| --- | --- |
| 1.1. Are the sources of qualitative data (archives, documents, informants, observations) relevant to address the research question (objective)? |  |
| 1.2. Is the process for analyzing qualitative data relevant to address the research question (objective)? |  |
| 1.3. Is appropriate consideration given to how findings relate to the context, e.g., the setting, in which the data were collected? |  |
| 1.4. Is appropriate consideration given to how findings relate to researchers’ influence, e.g., through their interactions with participants? |  |
| **2. Quantitative randomized controlled (trials)** |  |
| 2.1. Is there a clear description of the randomization (or an appropriate sequence generation)? |  |
| 2.2. Is there a clear description of the allocation concealment (or blinding when applicable)? |  |
| 2.3. Are there complete outcome data (80% or above)? |  |
| 2.4. Is there low withdrawal/drop-out (below 20%)? |  |
| **3. Quantitative non randomized** |  |
| 3.1. Are participants (organizations) recruited in a way that minimizes selection bias? |  |
| 3.2. Are measurements appropriate (clear origin, or validity known, or standard instrument; and absence of contamination between groups when appropriate) regarding the exposure/intervention and outcomes? |  |
| 3.3. In the groups being compared (exposed vs. non-exposed; with intervention vs. without; cases vs. controls), are the participants comparable, or do researchers take into account (control for) the difference between these groups? |  |
| 3.4. Are there complete outcome data (80% or above), and, when applicable, an acceptable response rate (60% or above), or an acceptable follow-up rate for cohort studies (depending on the duration of follow-up)? |  |
| **4. Quantitative descriptive** |  |
| 4.1. Is the sampling strategy relevant to address the quantitative research question (quantitative aspect of the mixed methods question)? |  |
| 4.2. Is the sample representative of the population understudy? |  |
| 4.3. Are measurements appropriate (clear origin, or validity known, or standard instrument)? |  |
| 4.4. Is there an acceptable response rate (60% or above)? |  |
| **5. Mixed methods** |  |
| 5.1. Is the mixed methods research design relevant to address the qualitative and quantitative research questions (or objectives), or the qualitative and quantitative aspects of the mixed methods question (or objective)? |  |
| 5.2. Is the integration of qualitative and quantitative data (or results*) relevant to address the research question (objective)? |  |
| 5.3. Is appropriate consideration given to the limitations associated with this integration, e.g., the divergence of qualitative and quantitative data (or results*) in a triangulation design? |  |
|  |  |
| **OVERALL SCORE (1* to 4*)** |  |

**SUPPLEMENTARY SEARCHES**

| **Papers in bibliography that may be relevant (out of how many?)**   - Out of how many? - **E.g. further use / evaluation of the software** - Papers that may be relevant can include systematic reviews and theoretical papers for the discussion section (not just papers that could be part of the synthesis) - Please read abstracts and titles of potentially relevant papers - Read the full paper if you think it may be relevant - List the title of full papers you read opposite - Highlight in bold full papers you have read that you think should be included - If you think a full paper should not be included after reading it, please put a brief reason why (ideally related to the inclusion / exclusion criteria detailed in the paper) |  |
| --- | --- |
| **Papers in citation searching that may be relevant (using Google Scholar)**   - Out of how many? - **E.g. further use / evaluation of the software** - Papers that may be relevant can include systematic reviews and theoretical papers for the discussion section (not just papers that could be part of the synthesis) - Please read abstracts and titles of potentially relevant papers - Read the full paper if you think it may be relevant - List the title of full papers you read opposite - Highlight in bold full papers you have read that you think should be included - If you think a full paper should not be included after reading it, please put a brief reason why (ideally related to the inclusion / exclusion criteria detailed in the paper) |  |
| **Papers in related article searching that may be relevant (Using Google Scholar – limit to first 100 results)**   - Out of how many? - **E.g. further use / evaluation of the software** - Papers that may be relevant can include systematic reviews and theoretical papers for the discussion section (not just papers that could be part of the synthesis) - Please read abstracts and titles of potentially relevant papers - Read the full paper if you think it may be relevant - List the title of full papers you read opposite - Highlight in bold full papers you have read that you think should be included - If you think a full paper should not be included after reading it, please put a brief reason why (ideally related to the inclusion / exclusion criteria detailed in the paper) |  |
