# Supplementary material for: Systematic review and narrative synthesis of computerized audit and feedback systems in healthcare
Source: J Am Med Inform Assoc. 2022 Mar 10;29(6):1106–19. doi: 10.1093/jamia/ocac031 (PMC9093027; doi:10.1093/jamia/ocac031)
Supplement: ocac031_Supplementary_Data [file ocac031_supplementary_data.zip › Additional File 3 (Summary of studies).docx]

# Additional file 3: Summary of included studies grouped by computerised audit and feedback (e-A&F) system

| **ID & citation** | **Country** | **Setting of study** | **Aim of study/research question** | **Study design** | **Methods** | **Key findings** | **Quality appraisal** |
| --- | --- | --- | --- | --- | --- | --- | --- |
| **141**  Waitman LR, Phillips IE, McCoy AB, Danciu I, Halpenny RM, Nelsen CL, Johnson DC, Starmer JM, Peterson JF. Adopting real-time surveillance dashboards as a component of an enterprise wide medication safety strategy. The Joint Commission Journal on Quality and Patient Safety. 2011 Jul 1;37(7):326-. | USA | Hospital care (single centre tertiary care hospital) | To design and implement a real-time surveillance application to enable pharmacy review of high-alert medication orders to complement existing systems | Mixed methods case study - system description and evaluation | System utilization was evaluated by examining user log data for each dashboard (warfarin, heparin and enoxaparin, and aminoglycoside antibiotics). To evaluate interventions made by the pharmacists, a qualitative analysis of comments generated for 100 patients from each dashboard was performed | Utilization patterns differed among the three dashboards. For warfarin, fewer than half of the cases required a detailed case review, but those cases were reviewed an average of four times each.  Even in an environment with computerized provider order entry and clinical decision support systems, real-time pharmacy surveillance of high-alert medications can intercept medication errors. | 1* |
| **282**  Boggan, Joel C., et al. A Novel Approach to Practice-Based Learning and Improvement Using a Web-Based Audit and Feedback Module. *Journal of graduate medical education* 6.3 (2014): 541-546.  **S2906**  Boggan JC, Swaminathan A, Thomas S, Simel DL, Zaas AK, Bae JG. Improving timely resident follow-up and communication of results in ambulatory clinics utilizing a web-based audit and feedback module. Journal of graduate medical education. 2017 Apr;9(2):195. | USA | Hospital care (single residency programme) | To examine whether an online tool for program-wide quality improvement initiatives result in improvement in diabetic foot examination compliance/quality | **282** - Before and after study  **S2906** - Before and after study | 282 - In phase 1, residents completed reviews of health records with online data entry. Residents were then presented with personal performance data relative to peers and were prompted to develop improvement plans. In phase 2, residents again reviewed personal performance.  S2906 - Residents performed audits of ambulatory patients requiring laboratory or radiologic testing by means of a shared online interface. The intervention consisted of an educational module viewed with initial audits, development of a personalized improvement plan after Phase 1, and repeated real-time feedback of individual relative performance compared at clinic and program levels. | 282 - Rates of performance of examination increased significantly between phases (from 52% to 73% for complete examination)^#^. 79 of 86 eligible residents (92%) completed improvement plans and reviewed 1471 patients in phase 1, whereas 68 residents (79%) reviewed 1054 patient charts in phase 2.  S2906 - Follow-up rates were higher in Phase 2 than Phase 1 for communicating results within 14 days and significant results within 72 hours (85% versus 78%, P < .001; and 82% versus 70%, P = .002, respectively). Communication of “significant” results was more likely to occur via telephone, compared with communication of non-significant results. | Both 2* |
| **400**  Tinoco A, Evans RS, Staes CJ, Lloyd JF, Rothschild JM, Haug PJ. Comparison of computerized surveillance and manual chart review for adverse events. Journal of the American Medical Informatics Association. 2011 Jul 1;18(4):491-7. | USA | Hospital care (single centre teaching hospital) | To understand how the source of information affects different adverse event (AE) surveillance methods | Retrospective cohort study | Retrospective analysis of inpatient adverse drug events (ADEs) and hospital-associated infections (HAIs) detected by either a computerized surveillance system(CSS) or manual chart review (MCR). | CSS detected more hospital-associated infections (HAIs) than manual chart review (92% vs 34%). A similar number of ADEs was detected by both systems (52% vs 51%). The agreement between systems was greater for HAIs than ADEs (26% vs 3%). | 3* |
| **417**  Carney PA, Abraham L, Cook A, Feig SA, Sickles EA, Miglioretti DL, Geller BM, Yankaskas BC, Elmore JG. Impact of an educational intervention designed to reduce unnecessary recall during screening mammography. Academic radiology. 2012 Sep 30;19(9):1114-20.  **418**  Carney PA, Bowles EJ, Sickles EA, Geller BM, Feig SA, Jackson S, Brown D, Cook A, Yankaskas BC, Miglioretti DL, Elmore JG. Using a tailored web-based intervention to set goals to reduce unnecessary recall. Academic radiology. 2011 Apr 30;18(4):495-503.  **1892**  Carney PA, Geller BM, Sickles EA, Miglioretti DL, Bowles EJ, Abraham L, Feig SA, Brown D, Cook AJ, Yankaskas BC, Elmore JG. Feasibility and satisfaction with a tailored web-based audit intervention for recalibrating radiologists’ thresholds for conducting additional work-up. Academic radiology. 2011 Mar 31;18(3):369-76. | USA | Hospital care (Radiology) | **417 -** To describe the impact of a tailored web based educational program designed to reduce excessive screening mammography recall  **418 –** To examine whether an intervention strategy consisting of a tailored web-based intervention, which provides individualized audit data with peer comparisons and other data that can affect recall can assist radiologists in setting goals for reducing unnecessary recall  **1892 –** To examine the feasibility of and satisfaction with a tailored web-based intervention designed to decrease radiologists' recommendation of inappropriate additional work-up following a screening mammogram. | **417 –**RCT  **418**f qualitative study **1892** qualitative study | The intervention provided each radiologist with individual audit data for their sensitivity, specificity, recall rate, PPV and cancer detection rates compared to national benchmarks and peer-comparisons for the same measures; profiled breast cancer risk in each radiologist’s respective patient populations to illustrate how low breast cancer risk is in population based settings, and evaluated the possible impact of medical malpractice concerns on recall rates. Participants’ recall rates from actual practice were evaluated for three time periods: the nine months before the intervention was delivered to the Intervention Group (baseline period), the nine months between the Intervention and Control Groups (T1) and nine months after Completion of the intervention by the Controls (T2) | **417** – (impact) the study resulted in a null effect, which may indicate a single one-hour intervention is not adequate to change excessive recall among radiologists who undertook the intervention we were testing  **418** (goal-setting) – the intervention does help goal setting:31 radiologists (72.1%) indicated they would like to change their recall rates and 30 (69.8%) entered a text response about changing their rates. Sixteen of the 30 (53.3%) radiologists who included a text response set realistic goals.  **1892 –** (Feasibility& satisfaction) only55% who consented to the study actually undertook the intervention, On average, three log-ins were used to complete the program (range 1–14), which took approximately 1 hour. Ninety-five percent found the program moderately to very helpful in understanding how to calculate basic performance measures. Ninety-three percent found viewing their own performance measures moderately to very helpful, and 83% reported it being moderately to very important to learn that the breast cancer risk in their screening population program was lower than perceived. | All 1* |
| **540**  Crits-Christoph P, Ring-Kurtz S, McClure B, Temes C, Kulaga A, Gallop R, Forman R, Rotrosen J. A randomized controlled study of a web-based performance improvement system for substance abuse treatment providers. Journal of substance abuse treatment. 2010 Apr 30;38(3):251-62. | USA | Hospital care (community-based outpatient substance abuse treatment clinics) | To investigate whether the use of the PF system would result in more positive outcomes with regard to greater improvements in average patient drug and alcohol use, attendance at group counselling sessions, and alliance. | Randomised controlled trial | 10 clinics received 12 weeks of the patient feedback performance improvement intervention, and 10 clinics received no intervention during the 12 weeks. More than 1,500 patients provided anonymous ratings of therapeutic alliance, treatment satisfaction, and drug/alcohol use. | No effect - There was no evidence of an intervention effect on the primary drug and alcohol use scales. There was also no evidence of an intervention effect on secondary measures of therapeutic alliance. Clinician-rated measures of organizational functioning and job satisfaction also showed no intervention effect. | 3* |
| **565**  Peiris D, Usherwood T, Panaretto K, Harris M, Hunt J, Redfern J, Zwar N, Colagiuri S, Hayman N, Lo S, Patel B. Effect of a Computer-Guided, Quality Improvement Program for Cardiovascular Disease Risk Management in Primary Health Care The Treatment of Cardiovascular Risk Using Electronic Decision Support Cluster-Randomized Trial. Circulation: Cardiovascular Quality and Outcomes. 2015 Jan 1;8(1):87-95 | Australia | Primary care (60 primary healthcare centres) | To test whether a multifaceted quality improvement intervention comprising computerized decision support, audit/feedback tools, and staff training improved:  1) guideline-indicated CVD risk factor measurements  2) guideline-indicated medications for those at high cardiovascular disease risk. | Parallel arm cluster-randomised controlled trial | Randomization was in a 1:1 allocation to the intervention or usual care stratified at 3 levels: (1) Aboriginal Community Controlled Health Services vs general practices; (2) service size (<500 patients vs ≥500); and (3)current participation in a national or state QI program Centres had to use a compatible software system, and eligible patients were regular attendees. Patient-level analyses were conducted using generalized estimating equations to account for clustering | The intervention results in a 10% absolute improvement in screening for cardiovascular disease risk. However, there were no significant improvements in prescribing recommended medicines to people at high cardiovascular disease risk although there were significant improvements in treatment escalation (new prescriptions or increased numbers of medicines).  Mean monthly staff support was <1 hour/site | 3* |
| **642**  Dixon‐Woods MA, Redwood S, Leslie M, Minion J, Martin GP, Coleman JJ. Improving quality and safety of care using technovigilance: an ethnographic case study of secondary use of data from an electronic prescribing and decision support system. Milbank Quarterly. 2013 Sep 1;91(3):424-54 | UK | Hospital care (a single large English acute care hospital with a well-established ePDSS) | To evaluate an approach to improving patients’ safety and quality of care involving the secondary use of data from a hospital electronic prescribing and decision support system (ePDSS). | Qualitative study (ethnography and interviews) | 162 hours were spent in ethnographic observation, including informal chats and conversations with front-line staff recorded as field notes, on four clinical areas (two wards, the pharmacy, and a specialist clinical unit within the hospital). 10semi structured interviews with hospital senior executives, were recorded using a digital recorder. Also 5 care omission meetings and eight additional meetings (28 hours) were observed | The hospital’s approach to quality and safety could be characterized as ‘technovigilance’.Through a combination of rapid audit, feedback to clinical teams, detailed and critical review of apparent omissions in executive-led meetings, a focus on personal professional responsibility for patients’ safety and quality care, and the correction of organizational or systems defects, technovigilance was—based on the hospital's own evidence—highly effective in improving specific indicators. Measures such as the rate of omitted doses of medication showed marked improvement. Technovigilance also had unintended consequences. These included the risk of focusing attention on aspects of patient safety made visible by the system at the expense of other, less measurable concerns. | 4* |
| **657**  Driessen SR, van Zwet EW, Haazebroek P, Sandberg EM, Blikkendaal MD, Twijnstra AR, Jansen FW. A Dynamic Quality Assessment Tool for Laparoscopic Hysterectomy to Measure Surgical Outcomes. Obstetrical &Gynecological Survey. 2017 Jan 1;72(1):18-32. | Netherlands | Hospital care (international multicentre) | To develop and test a quality assessment tool for laparoscopic hysterectomy, which can serve as a new outcome quality indicator | Prospective case report &qualitative study | A web-based application was developed with three main goals. All gynaecologists who perform laparoscopic hysterectomies were requested to register their procedures in the application. A patient safety risk factor checklist was used by the surgeon for reflection. Thereafter, a prospective implementation study was performed, and the application was tested using a survey that included the System Usability Scale. | The majority of surgeons reported that the application made them more aware of their performance, the outcomes, and patient safety, and they noted that the application provided motivation for improving future performance. (via a survey)  The overall survey response rate was 75%, and the mean System Usability Scale was 76.5 ± 13.6, which indicates that the application was good to excellent. | 3* |
| **715**  van Engen-Verheul MM, Gude WT, van der Veer SN, Kemps HM, Jaspers MM, de Keizer NF, Peek N. Improving guideline concordance in multidisciplinary teams: preliminary results of a cluster-randomized trial evaluating the effect of a web-based audit and feedback intervention with outreach visits. InAMIA Annual Symposium Proceedings 2015 (Vol. 2015, p. 2101). American Medical Informatics Association.  **947**  Gude WT, van Engen-Verheul MM, van der Veer SN, de Keizer NF, Peek N. How does audit and feedback influence intentions of health professionals to improve practice? A laboratory experiment and field study in cardiac rehabilitation. BMJ QualSaf. 2017 Apr 1;26(4):279-87.  **948**  Gude WT, van der Veer S, van Engen-Verheul M, de Keizer N, Peek N. Inside the Black Box of Audit and Feedback: a Laboratory Study to Explore Determinants of Improvement Target Selection by Healthcare Professionals in Cardiac Rehabilitation. In MedInfo 2015 Aug 12 (pp. 424-428).  **S2904**  Gude WT, van Engen-Verheul MM, van der Veer SN, Kemps HM, Jaspers MW, de Keizer NF, Peek N. Effect of a web-based audit and feedback intervention with outreach visits on the clinical performance of multidisciplinary teams: a cluster-randomized trial in cardiac rehabilitation. Implementation Science. 2016 Dec 9;11(1):160. | Netherlands | Hospital care (multicentre 18 cardiac rehab (CR) clinics) | **715** - To assess the effectiveness of the multi-faceted intervention  **947** - To identify factors that influence the intentions of health professionals to improve their practice when confronted with clinical performance feedback, which is an essential first step in the audit and feedback mechanism  **948** - To determine how healthcare professionals select quality indicators as targets for improvement based on reports of performance feedback, and the reasons for disregarding feedback recommendations for selecting indicators  **2904** –to assess the effect of a web-based audit and feedback (A&F) intervention with outreach visits to support decision-making by multidisciplinary teams | **715** - Cluster-randomized trial (preliminary results)  **947**– Controlled laboratory experiment, and a field study  **948** – Survey/ end user testing  **2904** – Cluster-randomized trial (finished results) | **715** - Based on known facilitators of successful A&F interventions, a web-based A&F intervention was developed with indicator-based performance feedback, benchmark information, action planning and outreach visits. During these visits the system was used to guide the clinics’ local QI teams through the process of systematically defining, implementing and monitoring QI actions.  **947** –The controlled laboratory experiment was a secondary analysis of data on 14 847 patients from 18 centres collected during a cluster-randomised controlled trial. Feedback reports were provided through a web-based application, and included performance scores and benchmark comparisons (high, intermediate or low performance). From each report participants selected indicators for improvement into their action plan. Our unit of observation was an indicator presented in a feedback report (selected yes/no).  **948** –two real performance feedback reports were randomly selected from a report database containing all 50 reports that were previously generated for CR clinics participating in the trial. They were presented as if they concerned reports of a ‘virtual’ CR’ clinic. Respondents were asked to select the quality indicators that they thought should be improvement targets in the QI plan of the virtual clinic. To determine reasons for disregarding feedback’s recommendations for (not) selecting indicators as improvement targets, we asked respondents to explain their choice if they chose not to select an indicator with a red or yellow colour via survey.  **2904** – Teams were randomized either to receive feedback that was limited to psychosocial rehabilitation (study group A) or to physical rehabilitation (study group B). The intervention included web-based A&F with feedback on clinical performance, facilities for goal setting and action planning, and educational outreach visits. The main outcome measure was the difference in performance between study groups in 11 care processes and six patient outcomes, measured at patient level. Secondary outcomes included effects on guideline concordance for the four main CR therapies. | **715 –**No increase in concordance with guideline recommendations was observed.  **947 –**Each 10% decrease in performance score increased the probability of an indicator being selected by 54% in the laboratory experiment, and 25% in the field study. Also, performance being benchmarked as low and intermediate increased this probability in laboratory settings. Still, participants ignored the benchmarks in 34% and 48% respectively. The main reason for participants to select an indicator reflecting high performance was that they considered the indicator an essential aspect of CR care quality, and should thus belong in every quality improvement plan (n=27, 82%). All other reasons were selected in <10% of cases.  **948 –**There was no association between the indicator type nor any of the professional-level factors and the selection of an indicator as target for improvement. Professionals were more likely to select an indicator if there was more room for improvement, and they were more strongly influenced by the reported colour (red, yellow, green) than by the reported performance score. Still, they ignored more than a quarter of the feedback’s recommendations for (not) selecting indicators based on the reported colours. Indicators for which insufficient data were available to calculate a score (indicators with a grey colour) were always selected. In addition, the number of selected indicators varied strongly between individual participants.  **2904** –no effect of our intervention on any of our primary or secondary outcome measures. During the intervention, a total of 233 quality improvement goals was identified by participating teams, of which 49 (21%) were achieved during the study period. | All 2* |
| **724**  Estrada CA, Safford MM, Salanitro AH, Houston TK, Curry W, Williams JH, Ovalle F, Kim Y, Foster P, Allison JJ. A web-based diabetes intervention for physician: a cluster-randomized effectiveness trial. International Journal for Quality in Health Care. 2011 Dec 1;23(6):682-9.  **S2845**  Billue KL, Safford MM, Salanitro AH, Houston TK, Curry W, Kim Y, Allison JJ, Estrada CA. Medication intensification in diabetes in rural primary care: a cluster-randomised effectiveness trial. BMJ open. 2012 Jan 1;2(5):e000959. | USA | Primary care (multicentre across 11 US Southeastern states) | To determine the effectiveness of a provider-based education and implementation intervention for:  **724 –** improving diabetes control  **S2845 –**improving medication intensification among patients with diabetes. | Cluster-randomized trial – (724 and S2845 examined different sets of data) | Physicians were randomized equally to the intervention (intervention website, performance feedback reports & email reminders) or control (control website) arms. All participating physicians provided copies of records of 15 (intervention arm physicians) or 10 (control arm physicians) consecutively seen patients with diabetes at baseline and again at follow-up (representing two cross-sectional views of each physician’s practice). | **724 –** The wide-reach, low-intensity, Web-based interactive multi-component intervention did not improve control of glucose, BP or lipids for patients with diabetes. Physicians engaged with the website over a median of 64.7 weeks and 37 min.  **S2845 –** Medication intensification to control glucose increased in **both** the intervention and control groups and it was most significant at very poor levels of glucose control. (But no effect of the intervention alone) | Both 1* |
| **756**  Feldstein AC, Schneider JL, Unitan R, Perrin NA, Smith DH, Nichols GA, Lee NL. Health care worker perspectives inform optimization of patient panel-support tools: a qualitative study. Population health management. 2013 Apr 1;16(2):107-19.  **S2884**  Feldstein AC, Perrin NA, Unitan R, Rosales AG, Nichols GA, Smith DH, Schneider J, Davino CM, Zhou YY, Lee NL. Effect of a patient panel-support tool on care delivery. The American journal of managed care. 2010 Oct;16(10):e256-66. | USA | Primary care | **756 –T**o evaluate perspectives about using the Patient Panel-Support Tool (PST) to better understand health care workers' attitudes toward, and adoption and use of, a decision-support tool  **S2884 –**To evaluate the effect of a patient panel-support tool (PST) on care delivery for diabetes mellitus (DM) and cardiovascular disease (CVD). | **756**-Qualitative study  **S2884 -** Retrospective longitudinal cohort | **756 -**Primary care physician (PCP) teams were grouped (based on performance pre- and post-PST introduction) into lower, improving, and higher percent-of-care-needs met. 21 interviews were conducted to elicit participant perspectives about the PST  **S2884 –**We estimated the intervention effect using electronic medical record data and hierarchical linear models. Patients had DM or CVD and 12 months of membership (n = 30,273 DM; 26,414 CVD). Main measures were mean percentages of care recommendations that were met by PCPs per patient per month (the care score) | **756 -**the most commonly cited benefit of the PST was increased in-depth knowledge of patient panels, and empowerment of staff to do quality improvement. Barriers to PST use included insufficient time, competing demands, suboptimal staffing, tool navigation, documentation, and data issues. Facilitators were strong team staff roles, leadership/training for tool implementation, and dedicated time for tool use. Higher performing PCPs and their assistants more often described a detailed team approach to using the PST  **S2884 –**From 2005 to 2007, the mean care score increased for both DM and CVD, from 63.5 (62.7, 64.3) to 70.6 (69.8, 71.4) and from 67.9 (67.2, 68.7) to 72.6 (71.9, 73.3), respectively. After adjustments, DM and CVD patients had improvements in the care score of 7.6 and 5.1, respectively, in 2007 compared with 2005.^#^ Several other factors significantly affected the care scores for both patient cohorts. The PCPs with higher baseline care scores had smaller increases in the rate of monthly change from pre-PST to PST implementation period for both DM and CVD. | **756** – 3*  **S2884 –** 4* |
| **772**  Filardo G, Nicewander D, Herrin J, Edwards J, Galimbertti P, Tietze M, Mcbride S, Gunderson J, Collinsworth A, Haydar Z, Williams J. A hospital-randomized controlled trial of a formal quality improvement educational program in rural and small community Texas hospitals: one year results. International Journal for Quality in Health Care. 2009 Aug 1;21(4):225-32.  **773**  Filardo G, Nicewander D, Herrin J, Galimbertti P, Tietze M, McBride S, Gunderson J, Collinsworth A, Haydar Z, Williams J, Ballard DJ. Challenges in conducting a hospital-randomized trial of an educational quality improvement intervention in rural and small community hospitals. American Journal of Medical Quality. 2008 Nov;23(6):440-7. | USA | Hospital care (multicentre - 47 rural and small community Texas hospitals) | **772 –** To investigate the effectiveness of a quality improvement educational program in rural hospitals  **773 –** To describe some of the challenges that researchers encounter in implementing trials of quality improvement interventions in real-world settings. | Randomised control trial (Same study looking a different aspects) | The 47 hospitals were randomized either to receive formal quality improvement educational program or to a control group. Performance on core measures for community-acquired pneumonia and congestive heart failure were compared between study groups to evaluate the impact of the educational program | **772 –** No significant differences were observed between the study groups on any measures. Of the 23 hospitals in the intervention group, only 16 completed the didactic program and 6 the full training program. Similar results were obtained when these groups were compared with the control group.  **773 –** Of the 64 hospitals initially interested in participating, 7 could not produce the required quality data and 10 refused consent to randomization. Quality improvement interventions require several different approaches to engage participating organizations and should include plans to train new staff given the high turnover of health care quality improvement personnel. | Both 1* |
| **779**  Fisher JC, Godfried DH, Lighter-Fisher J, Pratko J, Sheldon ME, Diago T, Kuenzler KA, Tomita SS, Ginsburg HB. A novel approach to leveraging electronic health record data to enhance pediatric surgical quality improvement bundle process compliance. Journal of pediatric surgery. 2016 Mar 2. | USA | Hospital care | Increasing bundle compliance and reducing adverse events | Before and after study | Ten SSI prevention bundle processes were linked to EHR data elements that were then aggregated into a snapshot display superimposed on weekly case-log reports | Nine months after implementation of our custom EHR tool, we observed centerline shifts in median SSI bundle compliance (46% to 72%). Additionally, as predicted by high reliability principles, we began to see a trend toward improvement in SSI rates (1.68 to 0.87 per 100 operations), but a discrete centerline shift was not detected. | 2* |
| **875**  Geller BM, Ichikawa L, Miglioretti DL, Eastman D. Web-based mammography audit feedback. American Journal of Roentgenology. 2012 Jun;198(6):W562-7. | USA | Hospital care | To develop and test an interactive Website to provide screening and diagnostic mammography audit feedback with comparisons to national and regional benchmarks. | End user survey  35 Radiologists of varying experience – some over 20 years | Radiologists who participate in three Breast Cancer Surveillance Consortium registries in the United States were invited during 2009 and 2010 to use a Website that provides tabular and graphical displays of mammography audit reports with comparisons to national and regional performance measures. We collected data about the use and perceptions of the Website. | 35 of 111 invited radiologists used the Website from one to five times in a year. The most popular measure was sensitivity for both screening and diagnostic mammography, whereas a table with all measures was the most visited page. Of the 13 radiologists who completed the post-use survey, all found the Website easy to use and navigate, 11 found the benchmarks useful, and nine reported that they intended to improve a specific outcome measure that year. 13 (37%) completed a survey after using the Website. Nine reviewed their outcome data annually; two, every 6 months; and two, occasionally. | 1* |
| **950**  Guldberg TL, Vedsted P, Kristensen JK, Lauritzen T. Improved quality of Type 2 diabetes care following electronic feedback of treatment status to general practitioners: a cluster randomized controlled trial. Diabetic Medicine. 2011 Mar 1;28(3):325-32.  **951**  Guldberg TL, Vedsted P, Lauritzen T, Zoffmann V. Suboptimal quality of type 2 diabetes care discovered through electronic feedback led to increased nurse–GP cooperation. A qualitative study. primary care diabetes. 2010 Apr 1;4(1):33-9. | Denmark | Primary care (multicentre) | **950 –** To evaluate the effect of an electronic feedback system to general practitioners on quality of Type 2 diabetes care. **950**  **951**  To understand the influence of electronic feedback system (EFS) on providing type 2 diabetes care in general practice using a qualitative approach embedded in a randomized controlled trial. | **950**  Cluster randomised control trial  **951**  Qualitative study | **950 –** GPs were randomized to receive or not to receive electronic feedback on quality of care. Primary end-points were processes of care according to guidelines on prescriptions redeemed for Type 2 diabetes treatments, measuring of glycated haemoglobin and cholesterol and visits to ophthalmologists. Data were analysed using generalized linear models accounting for clustering at practice level.  **951**  13 GPs from 2 solo and 2 partnership practices were interviewed about their experience with the EFS which provided data on the quality of diabetes care in their practices. Data was analysed using a qualitative descriptive approach. | **950 – P**eople in the intervention group more often redeemed recommended prescriptions than people in the control group, respectively, as follows: oral antidiabetic treatment (32.8 vs. 12.0%)^#^, insulin treatment (33.8 vs. 12.4%)^#^, lipid-lowering medication (38.3 vs. 18.6%)^#^ and blood pressure medication (27.6 vs. 16.3%)^#^. There were no differences in mean glycated haemoglobin and serum cholesterol between the two groups.  **951**  All participants found the insight into the overall quality of their diabetes care beneficial. While the two solo practices reported of no changes, the EFS catalysed organisational changes in the 2 partnership practices by allocating a number of diabetes controls to nurses. Limited time and lack of real-time data were main barriers. | Both 2* |
| **973**  Michtalik HJ, Carolan HT, Haut ER, Lau BD, Streiff MB, Finkelstein J, Pronovost PJ, Durkin N, Brotman DJ. Use of provider‐level dashboards and pay‐for‐performance in venous thromboembolism prophylaxis. Journal of hospital medicine. 2015 Mar 1;10(3):172-8. | USA | Hospital care (single tertiary care centre) | To sequentially examine an individualized physician dashboard and pay-for-performance program to improve VTE prophylaxis rates amongst hospitalists. | Sequential before and after study | A Web-based hospitalist dashboard provided VTE prophylaxis feedback. After 6 months of feedback only, a pay-for-performance program was incorporated, with graduated payouts for compliance rates of 80-100%. | Monthly VTE prophylaxis compliance rates were 86%, 90%, and 94% during the baseline, dashboard, and combined dashboard/pay-for-performance periods, respectively. Compliance significantly improved with the use of the dashboard^#^ and addition of the pay-for-performance program^#^. The highest rate of improvement occurred with the dashboard (1.58%/month)^#^. | 2* |
| **1006**  Harris S, Morgan M, Davies E. Web-based reporting of the results of the 2006 four country prevalence survey of healthcare associated infections. Journal of Hospital Infection. 2008 Jul 31;69(3):258-64. | UK | Hospital care | To describe the development and operational details of the web-based reporting system used to report the 2006 prevalence survey data. | Case study | A web-based reporting system was developed in order to feed back the results of the 2006 prevalence survey of healthcare-associated infections in a timely manner to all participating hospitals in England, Wales and Northern Ireland. The database accommodated ∼75 000 records from over 250 hospitals. The reporting system was hosted on the National Health Service intranet, accessible via secure login. Users were able to access their individual Trust data via a series of predefined reports and an export facility was included to facilitate additional analysis.  A user satisfaction survey was undertaken approximately six months after the system launch | In total, 2448 visits were made to the reporting system. 48/151 infection control personnel responded to the questionnaire and >90% thought the accessibility of the system was good or satisfactory, easy to use, reliable and fast. Only 14% (7/48) stated that they used the export facility to carry out their own analysis. 83% (39/47) thought the report content was useful, the remainder found it unsatisfactory. The majority of users stated that their first choice for data feedback for future surveys would be via a web-based reporting system. | 1* |
| **1011**  Hartzler AL, Chaudhuri S, Fey BC, Flum DR, Lavallee D. Integrating patient-reported outcomes into spine surgical care through visual dashboards: lessons learned from human-centered design. eGEMs. 2015;3(2). | USA | Hospital care | To describe the human-centered design (HCD) framework and illustrate our application of HCD to guide participatory development of patient reported outcomes Dashboards with users, and share lessons learned and recommendations for future efforts to engage health care professionals in the design of user-friendly health information technology. | Case study | HCD methods were employed to create visual displays of patient reported outcomes (PRO) to support patient care and quality improvement. Through three major steps, health care professionals were engaged in targeted, iterative design activities to inform the development of a dashboard that visually displays patient-reported pain and disability outcomes following spine surgery. | Stakeholder feedback shaped user interface design features, and revealed important considerations for quality indicators and privacy-preserving sharing. Engaging health care professionals as stakeholders is a critical step toward the design of user-friendly system that is accepted, usable, and has the potential to enhance quality of care and patient outcomes. Users liked viewing trends over time and found the views to provide a useful basis for comparison.  Usability testing showed good task performance, reflected acceptability, and revealed a number of additional refinements for future improvements (e.g., information buttons and other context sensitive help, privacy preserving measures). | 2* |
| **1099**  Hysong SJ, Kell HJ, Petersen LA, Campbell BA, Trautner BW. Theory-based and evidence-based design of audit and feedback programmes: examples from two clinical intervention studies. BMJ quality & safety. 2017 Apr 1;26(4):323-34. | USA | Primary care (Multicentre) | To test the effect of explicit financial incentives to reward guideline-recommended hypertension care. | Four-arm cluster randomised control trial | A web-based, report-style feedback intervention was designed to help primary care physicians improve their care of patients with hypertension. Research assistants enrolled a minimum of five full-time primary care physicians from 12 hospital-based primary care clinics.These were randomized to one of four study groups: 1) physician-level (individual) incentives; 2) practice-level incentives; 3) physician-level plus practice-level (combined) incentives; and 4) no incentives (control). A random subset of 28 physicians was interviewed at 16-months. | There was an average 11% increase (SD=2.87) in use of guideline-recommended antihypertensive medications between periods 1 and 5; this included the control group, who only received A&F reports.^#^ Interview respondents reported finding the feedback reports useful, as they were more detailed and focused than the performance measure reports they normally receive as part of their regular work duties. | 2* |
| **1187**  Meijers JM, Halfens RJ, Mijnarends DM, Mostert H, Schols JM. A feedback system to improve the quality of nutritional care. Nutrition. 2013 Aug 31;29(7):1037-41. | Netherlands | Primary care (Within care homes) | To develop a feedback system that improves the translation of malnutrition performance data from the Dutch National Prevalence Measurement of Care Problems (LPZ) into relevant evidence- and practice-based interventions in care homes. | Case study – with user testing and engagement (interviews, focus groups and evaluation questionnaire) | The process consisted of two stages. The first was the development of a feedback system. Twenty-four interviews were held with health care professionals in care homes that participated in the LPZ to gain insight into needs regarding the translation of performance data into relevant improvement interventions. Subsequently, three multidisciplinary focus groups discussed how to develop a feedback system to deal with those needs. In the second stage, the feasibility of this system was evaluated via a questionnaire. | 47 respondents (50.5%) evaluated the new feedback system as feasible. The content and design were perceived to be very useful. Half of the participating institutions had already started working with improvement activities.  All participants indicated that the design of the dashboard led to an improved overview of nutrition performance data within the institution over the years and between institutions. Furthermore, it was much easier to see where improvement actions were needed. | 2* |
| **1219**  Jeffs L, Beswick S, Lo J, Lai Y, Chhun A, Campbell H. Insights from staff nurses and managers on unit-specific nursing performance dashboards: a qualitative study. BMJ quality & safety. 2014 Dec 1;23(12):1001-6. | Canada | Hospital care (single centre - teaching hospital) | To explore the perceptions and experiences of front-line nurses and managers associated with the implementation of a unit-level dashboard in one teaching hospital | Qualitative Interviews | A qualitative study was undertaken to explore the perceptions and experiences of front-line nurses and managers associated with the implementation of a unit-level dashboard. Six units were selected to participate in the study. Data were analysed using a directed content analysis approach. | Three key themes emerged around nurses’ and unit managers’ perspectives on the implementation of unit-specific dashboards. Nurses and managers described that the dashboard was a visual tool that displayed data on the impact of the nursing care provided to patients. This tool also was used by the nurses and managers to keep track of processes of care and patient outcomes and experiences at a unit level. Further, nurses were able to use performance data to identify quality care improvements specific to their unit. | 2* |
| **1472**  Linder JA, Schnipper JL, Tsurikova R, Yu DT, Volk LA, Melnikas AJ, Palchuk MB, Olsha-Yehiav M, Middleton B. Electronic health record feedback to improve antibiotic prescribing for acute respiratory infections. The American journal of managed care. 2010 Dec;16(12 Suppl HIT):e311-9. | USA | Primary care  (Multicentre) | To examine whether the Acute Respiratory Infection (ARI) Quality Dashboard, an electronic health record (EHR)–based feedback system, changed antibiotic prescribing | Cluster RCT | 27 primary care practices were randomly assigned to receive the Quality Dashboard or usual care. The primary outcome was the intent-to-intervene antibiotic prescribing rate. The antibiotic prescribing between ARI Quality Dashboard users and nonusers were also compared. | During the 9-month intervention, there was **no difference** between intervention and control practices in antibiotic prescribing for all ARI visits (47% vs 47%; P = .87), antibiotic-appropriate ARI visits (65% vs 64%; P = .68), or non–antibiotic-appropriate ARI visits (38% vs 40%; P = .70). Among the 258 intervention clinicians, 72 (28%) used the ARI Quality Dashboard at least once. These clinicians had a **lower overall ARI antibiotic prescribing rate** (42% vs 50% for nonusers)^#^. | 3* |
| **1478**  Lippert ML, Kousgaard MB, Bjerrum L. General practitioners uses and perceptions of voluntary electronic feedback on treatment outcomes–a qualitative study. BMC family practice. 2014 Nov 30;15(1):1.  **2252**  Schroll H, Thomsen JL, Andersen M, Friborg S, Søndergaard J. The danish model for improvement of diabetes care in general practice: impact of automated collection and feedback of patient data. International journal of family medicine. 2012 Jul 24;2012. | Denmark | Primary care  (Multicentre) | **1478** –to explore how and for what purposes indicator-based feedback is used by the general practitioners (GPs) and how they perceive it to contribute to their work.  **2252 –** Cohort study  To describe the development of the quality of care concerning drug prescriptions for diabetes patients listed with GPs using the Data Capture module | **1478** – Qualitative study (interviews)  **2252 –** Cohort study | **1478** – Qualitative interviews with nine GPs in two regions in Denmark. The main selection criterion was that the informants had experience with retrieving electronic feedback. The data generation was explorative and open-ended and the analysis took an iterative approach with continuous refinement of themes that emerged from the data.  **2252 –** We analyzed the change in the proportion of medication for uncontrolled cases of diabetes. using data collected automatically from GPs’ electronic health record system  Quality feedback reports are generated individually for each practice on the basis of the accumulated data and are available online only for the specific practice | **1478** – The study identified two main uses of feedback: i) Administration of a regular disease control schedule for patients with chronic disease and ii) Routine monitoring of outcomes for purposes of resource prioritisation and medication management. Both uses were deemed valuable by the GPs, but also as an additional extra to the clinical core task. All the GPs experienced the feedback to be of limited relevance to the most central and challenging aspects of clinical work understood as the care for individuals. This led to different reactions: Some GPs would use the feedback as a point of departure for broader deliberations about individual patient needs and treatment approaches. For others, the perceived limitations decreased their overall motivation to seek feedback.  **2252 –** From 2009 to 2010, there was an absolute risk reduction of 1.35% (0.89–1.81) in proportion of persons not in antidiabetic medication despite an HbA1c above 7.0.^#^ Similarly, there was a 4.51% (3.42–5.61) absolute risk reduction in patients not in antihypertensive treatment despite systolic blood pressure above 130 mm Hg and 4.73% (3.56–5.90: ) absolute risk reduction in patients with total cholesterol level above 4.5 mmol/L and not receiving lipid-lowering treatment.^#^ | **1478 –**2*  **2252 – 3*** |
| **1489**  Lo YS, Lee WS, Chen GB, Liu CT. Improving the work efficiency of healthcare-associated infection surveillance using electronic medical records. Computer methods and programs in biomedicine. 2014 Nov 30;117(2):351-9. | Taiwan | Hospital care (single tertiary-care teaching hospital) | To develop integrated healthcare-associated infection (HAI) surveillance information system, based on existing EHR systems for improving the work efficiency of infection control professionals (ICPs) and create a generic model as a common platform for comparing ICPs’ work efficiency. | Case study – task based analysis | To measure the system performance, we comparing the staff work efficiency when using two existing electronic culture-based surveillance information system (eCBSIS) and iHAUTISIS, respectively. A generic workflow model was created and demonstrates a patient's state (unsuspected, suspected, and confirmed) and corresponding time spent on surveillance tasks performed by ICPs for the patient in that state. | The study results showed that the iHAUTISIS performed better than the eCBSIS in terms of infection control professional’s time cost. It reduced the time by 73.27 s, when using iHAUTISIS (114.26 s) and eCBSIS (187.53 s), for each patient on average. | 2* |
| **1499**  Loo TS, Davis RB, Lipsitz LA, Irish J, Bates CK, Agarwal K, Markson L, Hamel MB. Electronic medical record reminders and panel management to improve primary care of elderly patients. Archives of internal medicine. 2011 Sep 26;171(17):1552-8. | USA | Primary care (single centre) | It was hypothesized that EMR reminders would improve adherence to practice guidelines and that benefits would be enhanced with the support of a panel manager. | Non-randomised control trial | We conducted a controlled trial to assess the effectiveness of electronic medical record (EMR) reminders, with or without panel management, on health care proxy designation, osteoporosis screening, and influenza and pneumococcal vaccinations in patients older than 65 years. Physicians were assigned to 1 of the following 3 arms: EMR reminder, EMR reminder plus panel manager, or control. We assessed completion of recommended practices during a 1-year period. | Electronic medical record reminders, when augmented by panel management, facilitated further improvement in all care areas examined, including vaccination rates and rates of health care proxy designation and bone density screening.^#^ | 2* |
| **1931**  Parsons A, McCullough C, Wang J, Shih S. Validity of electronic health record-derived quality measurement for performance monitoring. Journal of the American Medical Informatics Association. 2012 Jul 1;19(4):604-9. | USA | Primary care (Multicentre) | To determine the validity of EHR-derived quality measures and documented preventive services. | Cross sectional study | Retrospective electronic chart reviews of 4081 patient records across 57 practices were analyzed to determine the validity of EHR-derived quality measures and documented preventive services. | Workflow and documentation habits have a profound impact on EHR-derived quality measures. Compared with the manual review of electronic charts, EHR-derived measures can undercount practice performance, with a disproportionately negative impact on the number of patients captured as receiving a clinical preventive service or meeting a recommended treatment goal | 4* |
| **2003**  Pringle JL, Kearney SM, Grasso K, Boyer AD, Conklin MH, Szymanski KA. User testing and performance evaluation of the Electronic Quality Improvement Platform for Plans and Pharmacies. Journal of the American Pharmacists Association. 2015 Dec 31;55(6):634-41. | USA | Primary care (multicentre pharmacy chains) | To user-test and evaluate a performance information management platform that makes standardized, benchmarked medication use quality data available to both health plans and community pharmacy organizations. | Qualitative user evaluation study | During the first phase of the study, user experience was measured via user satisfaction surveys and interviews with key personnel (pharmacists, pharmacy leaders, and health plan leadership). Improvements were subsequently made to the platform based on these findings. During the second phase of the study, the platform was implemented in a greater number of pharmacies and by a greater number of payers. User experience was then re-evaluated to gather information for further improvements. | Users found the Web-based platform easy to use and beneficial in terms of understanding and comparing performance metrics. Primary concerns included lack of access to real-time data and patient-specific data. Many users also expressed uncertainty as to how they could use the information and data provided by the platform.  Future development is needed to ensure that the provided data can be transferred to pharmacy best practices and improved quality care. | 2* |
| **2180**  Redwood S, Ngwenya NB, Hodson J, Ferner RE, Coleman JJ. Effects of a computerized feedback intervention on safety performance by junior doctors: results from a randomized mixed method study. BMC medical informatics and decision making. 2013 Jun 4;13(1):1. | UK | Hospital care  (single teaching hospital) | To establish whether a junior doctor dashboard providing feedback on prescription warning information and laboratory alerting acceptance rates was effective in changing junior doctors’ behaviour. | Mixed methods approach – parallel group RCT, and individual and focus group interviews | A mixed methods approach was employed which included a parallel group randomised controlled trial, and individual and focus group interviews. Junior doctors below the specialty trainee level 3 grade were recruited and randomised to two groups. Every doctor (N = 42) in the intervention group was e-mailed a link to a personal dashboard every week for 4 months. Nineteen participated in interviews. The 44 control doctors did not receive any automated feedback. | No significant differences were observed in the rates of generating prescription warnings, or in the acceptance of laboratory alarms. However, responses to laboratory alerts differed between the pre-intervention and intervention periods. For the doctors of FY1 grade, this improvement was greater in the group with access to the dashboard (53.6% ignored pre vs 29.2% post intervention)^#^ than in the control group (47.9% ignored pre vs 47.0% post intervention). Qualitative interview data indicated that while junior doctors were positive about the electronic prescribing functions, they were discriminating in the way they responded to other alerts and warnings given that from their perspective these were not always immediately clinically relevant or within the scope of their responsibility. | 3* |
| **2201**  Hysong SJ, Knox MK, Haidet P. Examining clinical performance feedback in patient-aligned care teams. Journal of general internal medicine. 2014 Jul 1;29(2):667-74. | USA | Primary care (Multicentre) | To explore broad changes in clinical performance feedback associated with PACT implementation | Semi-structured interviews | Semi-structured interviews with primary care clinicians, their department heads, and facility leadership at 16 geographically diverse VA Medical Centers, selected purposively by their clinical performance profile. Interviews focused on how clinical performance information is fed back to clinicians, with particular emphasis on external peer-review program measures and changes in feedback associated with team-based care implementation. | Ownership of clinical performance still rests largely with the provider, despite transitioning to team-based care. A panel-management information tool emerged as the most prominent change to clinical performance feedback dissemination, and existing feedback tools were seen as most effective when monitored by the nurse members of the team. Facilities reported few, if any, appreciable changes to the assessment of clinical performance since transitioning to team-based care. | 2* |
| **2209**  Simon SR, Soumerai SB. Failure of Internet-based audit and feedback to improve quality of care delivered by primary care residents. International Journal for Quality in Health Care. 2005 Oct 1;17(5):427-31. | USA | Primary care  (Multicentre) | To determine the effectiveness of Internet-based audit and feedback to physicians to improve care for diabetes and hypertension. | Time-series analysis | We determined the proportion of each resident’s patients whose care fulfilled national guidelines for quality (i.e. diabetes patients had haemoglobin testing in the previous 6 months or hypertension patients received a β-blocker or diuretic in the same time period). The main outcome measures were (i) the proportion of resident physicians who accessed their profiles and (ii) change following the intervention in the proportion of patients whose care followed national guidelines. | Only four of the 12 residents accessed their websites. One of the residents visited her site three times, while the other three residents visited their sites once each. In interrupted time-series analyses, the intervention had no discernible effect on adherence to practice guidelines for diabetes or hypertension. | 2* |
| **2320**  Simpao AF, Ahumada LM, Desai BR, Bonafide CP, Gálvez JA, Rehman MA, Jawad AF, Palma KL, Shelov ED. Optimization of drug–drug interaction alert rules in a pediatric hospital's electronic health record system using a visual analytics dashboard. Journal of the American Medical Informatics Association. 2014 Oct 15:20(13) | USA | Hospital care (Tertiary care childrens’ hospital) | To develop and evaluate an electronic dashboard of hospital-wide electronic health record medication alerts for an alert fatigue reduction quality improvement project. | Cross-sectional study | We used visual analytics software to develop the dashboard. We collaborated with the hospital-wide Clinical Decision Support committee to perform three interventions successively deactivating clinically irrelevant drug–drug interaction (DDI) alert rules. We analyzed the impact of the interventions on care providers’ and pharmacists’ alert and override rates using an interrupted time series framework with piecewise regression. | The alert dashboard facilitated safe rapid-cycle reductions in alert burden that were temporally associated with lower pharmacist override rates in a subgroup of drug interactions not directly affected by the interventions;^#^ meanwhile, the pharmacists’ frequency of selecting the ‘cancel’ option increased.^#^ The medication serious safety event rate decreased during the study period, and there were no serious safety events reported in association with the deactivated alert rules. | 2* |
| **2616**  Ward CE, Morella L, Ashburner JM, Atlas SJ. An Interactive, All-Payer, Multidomain Primary Care Performance Dashboard. The Journal of ambulatory care management. 2014 Oct 1;37(4):339-48. | USA | Primary care (Multicentre) | To develop and implement a performance reporting system | Qualitative study | The case study describes an effort to engage local primary care providers (PCPs) as part of an internal process to develop and implement a performance reporting system. We developed and implemented an interactive, Web-based performance dashboard for primary care practices with input from provider focus groups. Adapting a business software application, individual physician and practice-level reports included information on visit-based and panel productivity, patient panel demographics, and outcome measures | Over 2 rounds of reporting, 69% to 77% of users viewed their report within 30 days and 79% of users found the report informative. | 3* |
| **2622**  Weber V, Bloom F, Pierdon S, Wood C. Employing the electronic health record to improve diabetes care: a multifaceted intervention in an integrated delivery system. Journal of general internal medicine. 2008 Apr 1;23(4):379-82. | USA | Primary care  (Multicentre) | To employ an electronic registry derived from a fully integrated electronic health record as the cornerstone of an intervention to improve compliance with recommended diabetes performance measures in an integrated practice network. | Before and after study | A multidisciplinary group of physicians worked to create a bundle of best practice measures for diabetes. This measurement tool was then used as part of a multifaceted intervention to improve physician performance in diabetes care, including audit and feedback, computerized reminders, and financial incentives. Changes in performance of individual measures and the total bundle were tracked monthly over 1 year. | Significant increases were seen in all measures of diabetes care over the 12-month period of the study. Vaccination for pneumococcal disease and influenza improved from 56.5% to 80.8%^#^ and 55.1% to 71.0%^#^. The percentage of patients with ideal glucose control (HBA1c < 7.0) increased from 32.2% to 34.8%# and blood pressure control (<130/80) improved from 39.7% to 43.9%^#^. The overall number of patients receiving all 9 bundled measurements improved from 2.4% to 6.5%^#^. | 3* |
| **S2838**  Blacky A, Mandl H, Adlassnig KP, Koller W. Fully automated surveillance of healthcare-associated infections with MONI-ICU. Applied clinical informatics. 2011;2(3):365-72. | Austria | Hospital care  (Single ICU unit) | To evaluate the accuracy and efficiency of the MONI-ICU system regarding the surveillance of healthcare-associated infections | Prospective longitudinal cohort | We describe the use of this system in clinical routine and compare the results generated automatically by MONI-ICU with those generated in parallel by trained surveillance staff using patient chart reviews and other available information (gold standard) | A total of 99 ICU patient admissions representing 1007 patient days were analyzed. MONI-ICU identified correctly the presence of an HCAI condition in 28/31 cases (sensitivity, 90.3%) and their absence in 68/68 of the non-HCAI cases (specificity, 100%), the latter meaning that MONI-ICU produced no false alarms. The 3 missed cases were due to correctable technical errors. The time taken for conventional surveillance at the 52 ward visits was 82.5 hours. MONI-ICU analysis of the same patient cases, including careful review of the generated results, required only 12.5 hours (15.2%). | 3* |
| **S2839**  Kilbridge PM, Noirot LA, Reichley RM, Berchelmann KM, Schneider C, Heard KM, Nelson M, Bailey TC. Computerized surveillance for adverse drug events in a pediatric hospital. J Am Med Inform Assoc. 2009 Sep-Oct;16(5):607-12. | USA | Hospital care (Tertiary teaching hospital) | To describe the implementation of an automated ADE detection system at a large pediatric hospital, and data from the operation of the system over a six month period. | Case study | The automated surveillance system screened admissions to a large pediatric hospital. Potential ADEs identified by the system were reviewed by medication safety pharmacists and a physician and scored for causality and severity. Over the 6 month study period, 6,889 study children were admitted to the hospital for a total of 40,250 patient-days. We describe our findings regarding the utility of different rule categories for event detection, and our overall findings of ADE rates in our pediatric population. | The ADE surveillance system generated 1226 alerts, which yielded 160 true ADEs. This represents a rate of 2.3 ADEs per 100 admissions or 4 per 1,000 patient-days. Medications most frequently implicated were diuretics, antibiotics, immunosuppressants, narcotics, and anticonvulsants. The composite positive predictive value of the ADE surveillance system was 13%. Automated surveillance can be an effective method for detecting ADEs in hospitalized children. | 2* |
| **S2847**  Hermon A, Pain T, Beckett P, Jerrett H, Llewellyn N, Lawrence P, Szakmany T. Improving compliance with central venous catheter care bundles using electronic records. Nursing in critical care. 2015 Jul 1;20(4):196-203. | UK (Wales) | Hospital care (single ICU in district hospital) | To describe the use of an electronic tool to monitor and feedback process compliance in conjunction of introducing bespoke central line insertion packs to tackle catheter-related bloodstream infections in the intensive care unit. | Quasi-experimental before and after | The central venous catheter insertion and maintenance bundle was rolled out in 2007. To monitor compliance, an electronic tool was designed as part of our bedside Clinical Information System. From 2009, regular quarterly feedback was provided on the number of central venous catheter lines inserted, compliance with the insertion and maintenance bundle and catheter-related bloodstream infection rate using the data collected through the system. Dedicated line insertion trolleys and factory-prepared insertion packs were also introduced. Segmented regression analysis was used to assess the changes in the catheter-related bloodstream infection rate before and after implementation of the central venous catheter bundle. | Bundle compliance increased during the implementation period and reached over 95% within 6 months. We observed a significant reduction in the catheter-related bloodstream infection rate from 15·6/1000 days to 0·4/1000 days.^#^ Regression analysis showed that only the compliance had significant effect on the number and prevalence of catheter-related bloodstream infections. | 1* |
| **S2863**  Kern LM, Malhotra S, Barron Y, Quaresimo J, Dhopeshwarkar R, Pichardo M, Edwards AM, Kaushal R. Accuracy of Electronically Reported Meaningful Use Clinical Quality Measures: A Cross-sectional Study. Annals of internal medicine. 2013 Jan 15;158(2):77-83. | USA | Primary care (multicentre) | To measure the accuracy of electronic reporting for 12 quality measures compared with manual review in a community-based setting. | Cross sectional study | This study compared electronic and manual chart documentation for 12 process-of-care measures in a single medical system. Sensitivity, specificity, positive and negative predictive values, positive and negative likelihood ratios, and absolute rates of recommended care were measured. | The accuracy of electronic reporting was highly variable overestimating some measures and underestimating others. Differences between electronic reporting and manual review were statistically significant for 3 measures: Electronic reporting underestimated the absolute rate of recommended care for 2 measures (appropriate asthma medication [38% vs. 77%]^#^ and pneumococcal vaccination [27% vs. 48%]^#^) and overestimated care for 1 measure (cholesterol control in patients with diabetes [57% vs. 37%]^#^). | 3* |
| **S2883**  McCoy AB, Cox ZL, Neal EB, Waitman LR, Peterson NB, Bhave G, Siew ED, Danciu I, Lewis JB, Peterson JF. Real-time pharmacy surveillance and clinical decision support to reduce adverse drug events in acute kidney injury: a randomized, controlled trial. ApplClin Inform. 2012 Jan 1;3(2):221-38. | USA | Hospital care (single tertiary teaching hospital) | To determine whether pharmacy surveillance of Acute Kidney Injury patients could detect and prevent medication errors that are not corrected by automated interventions. | Prospective (parallel group) RCT | The authors investigated 396 patients admitted with an acute 0.5 mg/dl change in serum creatinine over 48 hours and a nephrotoxic or renally cleared medication order. Patients randomly assigned to the intervention group received surveillance from a clinical pharmacist using a web-based surveillance tool to monitor drug prescribing and kidney function trends. CDS alerting and standard pharmacy services were active in both study arms.  Outcome measures included blinded adjudication of potential adverse drug events, adverse drug events and time to provider modification or discontinuation of targeted nephrotoxic or renally cleared medications. | Pharmacy surveillance had no incremental benefit over previously implemented CDS alerts. Potential ADEs or ADEs occurred for 104 (8.0%) of control and 99 (7.1%) of intervention patient-medication pairs (p=0.4). Additionally, the time to provider modification or discontinuation of targeted nephrotoxic or renally cleared medications did not differ between control and intervention patients (33.4 hrs vs. 30.3hrs). | 3* |
| **S2890**  Thomas KG, Thomas MR, Stroebel RJ, McDonald FS, Hanson GJ, Naessens JM, Huschka TR, Kolars JC. Use of a registry-generated audit, feedback, and patient reminder intervention in an internal medicine resident clinic—a randomized trial. Journal of general internal medicine. 2007 Dec 1;22(12):1740-4. | USA | Hospital care  (single centre) | To assess the effects of a registry-generated audit, feedback, and patient reminder intervention on diabetes care. | Prospective RCT | Residents randomized to the intervention (n = 39) received instruction on diabetes registry use; quarterly performance audit, feedback, and written reports identifying patients needing care; and had letters sent quarterly to patients needing hemoglobin A1c or cholesterol testing. Residents randomized to the control group (n = 39) received usual clinic education. | Patients cared for by residents in the intervention group had higher adherence to guideline recommendations for hemoglobin A1c testing (61.5% vs 48.1%, p = .01) and LDL testing (75.8% vs 64.1%, p = .02). Intermediate clinical outcomes were not different between groups. | 3* |
| **S2900**  Jonah L, Pefoyo AK, Lee A, Hader J, Strasberg S, Kupets R, Chiarelli AM, Tinmouth J. Evaluation of the effect of an audit and feedback reporting tool on screening participation: The Primary Care Screening Activity Report (PCSAR). Preventive medicine. 2017 Mar 31;96:135-43. | Canada | Primary care  (multicentre) | To evaluate the effectiveness of the 2014 PCSAR on screening participation in Ontario's three screening programs (breast, cervix and colorectal). | Retrospective cohort study | This study evaluated the effectiveness of the 2014 PCSAR on screening participation in Ontario’s three screening programs (breast, cervix and colorectal). Two exposures were evaluated for each cohort: enrolment with a physician who was registered to receive the PCSAR and enrolment with a registered physician who also logged into the PCSAR. Logistic regression modelling was used to assess the magnitude of the effect of PCSAR on participation, adjusting for participant and physician characteristics. | Across all three screening programs, 63% of eligible physicians registered to receive the PCSAR and 38% of those registered logged-in to view it. Patients of physicians who registered were significantly more likely to participate in screening, with odds ratios ranging from 1.06 to 1.15. The adjusted odds ratios associated with PCSAR log-in were 1.07 to 1.18 across all screening programs. Implementation of the PCSAR was associated with a small increase in screening participation. The PCSAR appears to be modestly effective in assisting primary care physicians in optimizing cancer screening participation among their patients. | 3* |
| **S2901**  Dreischulte T, Donnan P, Grant A, Hapca A, McCowan C, Guthrie B. Safer prescribing—a trial of education, informatics, and financial incentives. New England Journal of Medicine. 2016 Mar 17;374(11):1053-64.  **S2902**  Grant A, Dreischulte T, Guthrie B. Process evaluation of the data-driven quality improvement in primary care (DQIP) trial: active and less active ingredients of a multi-component complex intervention to reduce high-risk primary care prescribing. Implementation Science. 2017 Jan 7;12(1):4.  **S2903**  Grant A, Dreischulte T, Guthrie B. Process evaluation of the Data-driven Quality Improvement in Primary Care (DQIP) trial: case study evaluation of adoption and maintenance of a complex intervention to reduce high-risk primary care prescribing. BMJ open. 2017 Mar 1;7(3):e015281 | UK - Scotland | Primary care  (multicentre) | **S2901 -** whether the rates of high-risk prescribing by primary care clinicians and the related clinical outcomes would be reduced by a complex intervention.  **S2902 -** to explore response to the intervention delivered to clusters in relation to participants’ perceptions about which intervention elements were active in changing their practice  **S2903 -** To explore how different practices responded to the Data-driven Quality Improvement in Primary Care (DQIP) intervention in terms of their adoption of the work, re-organisation to deliver the intended change in care to patients, and whether implementation was sustained over time. | **S2901 –** Cluster -randomized, stepped-wedge trial  **S2902 –** Qualitative study  **S2903 -** Mixed-methods parallel process evaluation of a cluster trial, reporting the comparative case study of purposively selected practices. (qualitative arm looking at adoption of the work) | **S2901 -** Participating primary care practices were randomly assigned to various start dates for a 48-week intervention comprising professional education, informatics to facilitate review, and financial incentives for practices to review patients’ charts to assess appropriateness. Analyses were performed according to the intention-to-treat principle, with the use of mixed-effect models to account for clustering in the data.  **S2902 -** Data generation was by in-depth interview with key staff exploring participant’s perceptions of the intervention components. Analysis was iterative using the framework technique and drawing on normalisation process theory to examine the implementation of selected processes and their associations with change in high-risk prescribing at practice level.The qualitative element consisted of case studies in 10 of the 33 participating practices.  **S2903 -** We employed the use of qualitative data from interviews and observational field notes, and quantitative data about key trial processes and practice-level effectiveness to examine implementation in detail.  The evaluation sampled four practices which rapidly implemented the intervention and all six practices which failed to implement the intervention to some degree. | **S2901 -** Targeted high-risk prescribing was significantly reduced, from a rate of 3.7% immediately before the intervention to 2.2%^#^. The rate of hospital admissions for gastrointestinal ulcer or bleeding was significant from 55.7 to 37.0 admissions per 10,000 person-years,^#^ as was the rate of admissions for heart failure (from 707.7 to 513.5 admissions per 10,000 person-years)^#^, but admissions for acute kidney injury were not (101.9 and 86.0 admissions per 10,000 person-years)  **S2902 -** All the primary components of the intervention were perceived as active, but at different stages of implementation: financial incentives primarily supported recruitment; education motivated the GPs to initiate implementation; the informatics tool facilitated sustained implementation. Participants perceived the primary components as interdependent. Intervention subcomponents also varied in whether and when they were active. For example, run charts providing feedback of change in prescribing over time were ignored in the informatics tool, but were motivating in some practices in the regular e-mailed newsletter. The high-risk NSAID and anti-platelet prescribing targeted was accepted as important by all interviewees, and this shared understanding was a key wider context underlying intervention effectiveness.  **S2903 -** Four practices were sampled because they had large rapid reductions in targeted prescribing. They all had internal agreement that the topic mattered, made early plans to implement including assigning responsibility for work and regularly evaluated progress. However, how they internally organised the work varied.  Six practices were sampled because they had initial implementation failure. Implementation failure occurred at different stages depending on practice context, including internal disagreement about whether the work was worthwhile, and intention but lack of capacity to implement or sustain implementation due to unfilled posts or sickness.  All interviewed participants valued the intervention because it was an innovative way to address on an important aspect of safety (although one of the non-interviewed general practitioners in one practice disagreed with this). Participants felt that reviewing existing prescribing did influence their future initiation of targeted drugs, but raised concerns about sustainability. | All **3*** |
| S2905  Swartz J, Koziatek C, Theobald J, Smith S, Iturrate E. Creation of a simple natural language processing tool to support an imaging utilization quality dashboard. International Journal of Medical Informatics. 2017 May 31;101:93-9. | USA | Hospital care | 1) To develop and implement a simple, user-configurable, and open-source natural language processing tool to classify radiology reports with high accuracy  2) To use the results of the tool to design a provider-specific VTE imaging dashboard, consisting of both utilization rate and diagnostic yield. | Cross sectional study | Two physicians reviewed a training set of 400 lower extremity ultrasound (UTZ) and computed tomography pulmonary angiogram (CTPA) reports to understand the language used in VTE-positive and VTE-negative reports. The insights from this review informed the arguments to the five modifiable parameters of the NLP tool. A validation set of 2,000 studies was then independently classified by the reviewers and by the tool; the classifications were compared and the performance of the tool was calculated. | The tool was highly accurate in classifying the presence and absence of VTE for both the UTZ (sensitivity 95.7%; 95% CI 91.5–99.8, specificity 100%; 95% CI 100–100) and CTPA reports (sensitivity 97.1%; 95% CI 94.3–99.9, specificity 98.6%; 95% CI 97.8–99.4). The diagnostic yield was then calculated at the individual provider level and the imaging dashboard was created. | 2* |
| **S2907**  Trinh LD, Roach EM, Vogan ED, Lam SW, Eggers GG. Impact of a quality-assessment dashboard on the comprehensive review of pharmacist performance. Am J Health Syst Pharm. 2017 Sep 1;74(17 Supplement 3):S75-S83 | USA | Hospital care | The impact of a quality-assessment dashboard and individualized pharmacist performance feedback on the adherence of order verification was evaluated. | Before-and-after study | A total of 5 medications were assessed by the formulas for adherence and displayed on the dashboard: ampicillin–sulbactam, ciprofloxacin, piperacillin–tazobactam, acyclovir, and enoxaparin. Adherence of order verification was assessed before and after individualized performance feedback was given based on trends identified by the quality-assessment dashboard. | There was a significant increase in the overall adherence rate postintervention (90.1% versus 91.9%, p = 0.040). Among the 34 pharmacists who participated, time to verification was similar before and after the study intervention (median, 6.0 minutes; interquartile range, 3–13 minutes). The rate of documentation for nonadherent orders increased significantly postintervention (57.1% versus 68.5%, p = 0.019). | 2* |
| **S2908**  Bunce AE, Gold R, Davis JV, Mercer M, Jaworski V, Hollombe C, Nelson C. “Salt in the Wound”: safety net clinician perspectives on performance feedback derived from EHR data. The Journal of ambulatory care management. 2017 Jan;40(1):26.  **S2908B**  Gold R, Nelson C, Cowburn S, Bunce A, Hollombe C, Davis J, Muench J, Hill C, Mital M, Puro J, Perrin N. Feasibility and impact of implementing a private care system’s diabetes quality improvement intervention in the safety net: a cluster-randomized trial. Implementation Science. 2015 Dec;10(1):1-1. | USA | Primary Care | **S2908** – To better understand barriers to the perceived legitimacy of EHR-based feedback measures in primary care  **S2908B** - To identify and resolve barriers to effectively implementing a successful, privately developed QI program into primary care | **S2908** – Qualitative study  **S2908B** – Cluster randomised trial | **S2908** – Ethnographic observation, (126 field notes), semistructured interviews (34 transcripts), Group discussions (8 transcripts), Diaries by site co-ordinators, document and chart reviews  **2908B** - We conducted a cluster-randomized trial in 11 CHCs in a staggered process with six “early” CHCs implementing the intervention one year before five “‘late” CHCs. We measured monthly rates of patients with DM currently prescribed angiotensin converting enzyme (ACE)-inhibitors/statins, if clinically indicated. Through segmented regression analysis, we evaluated the intervention’s effects in June 2011–May 2013. | **S2908 –** Providers said EHR performance measures rarely accounted for CHC patients' complex lives or for providers' decisions as informed by this complexity, which diminished the measures' perceived validity.  **S908B –** One year post-implementation, in the early clinics, there were estimated relative increases in guideline-concordant prescribing of 37.6 % (95 % confidence interval (CI); 29.0–46.2 %) among patients indicated for both ACE-inhibitors and statins and 38.7 % (95 % CI; 23.2–54.2 %) among patients indicated for statins. No such increases were seen in the late (control) clinics in that period. This proved feasible and had significant impact but required considerable adaptation and implementation support. | Both 3* |
| **2909**  Banerjee D, Thompson C, Kell C, Shetty R, Vetteth Y, Grossman H, DiBiase A, Fowler M. An informatics-based approach to reducing heart failure all-cause readmissions: the Stanford heart failure dashboard. Journal of the American Medical Informatics Association. 2017 May 1;24(3):550-5. | USA | Hospital care | To test whether a comprehensive EMR-based approach utilizing an HF dashboard in addition to an established HF readmission reduction program would further reduce 30-day all-cause index hospital readmission rates for HF | Before and after study. | We instituted EMR-based measures designed to improve cohort identification, intervention tracking, and readmission analysis, the latter 2 supported by an electronic HF dashboard. Our primary outcome measure was the 30-day index hospital readmission rate for HF, with secondary measures including the accuracy of identification of patients with HF and the percentage of patients receiving interventions designed to reduce all-cause readmissions for HF. | The HF dashboard facilitated improved penetration of our interventions and reduced readmission rates. Implementation of our EMR-based approach further significantly reduced 30-day index hospital readmission rates for HF to 10.1% (P for trend = .0001). Daily time to screen patients decreased from 1 hour to 15 minutes, accuracy of cohort identification improved from 83% to 94.6% (P = .0001), and the percentage of patients receiving our interventions, such as patient education, also improved significantly from 22% to 100% over time | 1* |
| **2910**  Fletcher GS, Aaronson BA, White AA, Julka R. Effect of a real-time electronic dashboard on a rapid response system. Journal of medical systems. 2017 Jan;42(1):1-0. | USA | Hospital care | To evaluate the impact of an electronic medical record (EMR)-based alerting dashboard on outcomes associated with Rapid response Team (RRT) activation | Repeated treatment design study | We used a repeated treatment study in which the dashboard display was successively turned on and off each week for ten 2-week cycles over a 20-week period on the inpatient acute care wards of an academic medical center. | There were 6736 eligible admissions during the 20-week study period. There was no change in overall RRT activations (IRR = 1.14, p = 0.07), but a significant increase in first RRT activations (IRR = 1.20, p = 0.04). There were no significant differences in unexpected ICU transfers (IRR = 1.15, p = 0.25), cardiopulmonary arrests on general wards (IRR = 1.46, p = 0.43), or deaths on general wards (IRR = 0.96, p = 0.89). | 3* |
| **2911**  Lin LA, Bohnert AS, Kerns RD, Clay MA, Ganoczy D, Ilgen MA. Impact of the Opioid Safety Initiative on opioid-related prescribing in veterans. Pain. 2017 May 1;158(5):833-9. | USA | Primary and Hospital care | To examine changes associated with Opioid Safety Initiative (OSI) implementation in October 2013 among all adult VHA patients who filled outpatient opioid prescriptions. | Retrospective interrupted time series analysis | Interrupted time series analyses controlled for baseline trends and examined data from October 2012 to September 2014 to determine the changes after OSI implementation in prescribing of high-dosage opioid regimens (total daily dosages >100 morphine equivalents [MEQ] and >200 MEQ) and concurrent benzodiazepines. | Across VHA facilities nationwide, the OSI was associated with an additional decrease, compared to pre-OSI trends, of 331 patients per month (95% confidence interval [CI] −378 to −284) receiving opioids >100 MEQ, a decrease of 164 patients per month (95% CI −186 to −142) receiving opioids >200 MEQ, and a decrease of 781 patients per month (95% CI −969 to −593) receiving concurrent benzodiazepines. | 3* |
| **2912**  Kurtzman G, Dine J, Epstein A, Gitelman Y, Leri D, Patel MS, Ryskina K. Internal medicine resident engagement with a laboratory utilization dashboard: mixed methods study. Journal of hospital medicine. 2017 Sep;12(9):743.  **2912b**  Ryskina K, Dine CJ, Gitelman Y, Leri D, Patel M, Kurtzman G, Lin LY, Epstein AJ. Effect of social comparison feedback on laboratory test ordering for hospitalized patients: a randomized controlled trial. Journal of general internal medicine. 2018 Oct;33(10):1639-45. | USA | Hospital care | 2912 – To measure internal medicine resident engagement with an EMR-based dashboard providing continuous feedback on their use of routine laboratory tests relative to service averages.  2912B – To test the effect of such feedback on the ordering of routine laboratory tests for hospitalized patients, a practice considered overused. | 2912 – Mixed methods study  2912b – RCT | 2912 – From January to June 2016, residents were emailed a snapshot of their personalized dashboard, a link to the online dashboard, and text summarizing the resident’s and service utilization averages. We measured resident engagement using email read-receipts and web-based tracking. We also conducted three hour-long focus groups with residents. Using grounded theory approach, the transcripts were analyzed for common themes focusing on barriers and facilitators of dashboard use.  2912b – Physicians on six general medicine teams at the Hospital of the University of Pennsylvania were cluster randomized with equal allocation to two arms: (1) those e-mailed a summary of their routine laboratory test ordering vs. the service average for the prior week, linked to a continuously updated personalized dashboard containing patient-level details, and snapshot of the dashboard and (2) those who did not receive the intervention. The primary outcome was the count of routine laboratory test orders placed by a physician per patient-day. | 2912 – Among 80 residents, 74% opened the email containing a link to the dashboard and 21% accessed the dashboard itself. Residents who deviated further from service averages had significantly higher odds of accessing the dashboard.  2912b – One hundred and fourteen interns and residents participated. We did not observe a statistically significant difference in adjusted reduction in routine laboratory ordering between the intervention and control physicians (physicians in the intervention group ordered 0.14 fewer tests per patient-day than physicians in the control group, 95% CI − 0.56 to 0.27, p = 0.50). | 2912 – 2*  2912b – 1* |
| **S2913**  Jeffries M, Phipps DL, Howard RL, Avery AJ, Rodgers S, Ashcroft DM. Understanding the implementation and adoption of a technological intervention to improve medication safety in primary care: a realist evaluation. BMC health services research. 2017 Dec;17(1):1-1.  **S2914**  Jeffries M, Phipps D, Howard RL, Avery A, Rodgers S, Ashcroft D. Understanding the implementation and adoption of an information technology intervention to support medicine optimisation in primary care: qualitative study using strong structuration theory. BMJ open. 2017 May 1;7(5). | UK | Primary care | **S2913 –** To examine how an electronic medicines optimisation system was used for medicines safety activities in a primary care setting.  **S2914 –** To understand the adoption and implementation of an electronic clinical audit and feedback tool to support medicine optimisation for patients in primary care | **S2913 –** Qualitative realist evaluation  **S2914 –** Qualitative study | **S2913 –** Five semi-structured interviews, four focus groups and one observation were conducted with a range of stakeholders.  **S2914 –** Four focus groups and five semi-structured interviews were conducted with 18 participants purposively sampled from a range of stakeholder groups | **S2913 –** Using the electronic medicines optimisation system could lead to a number of improved patient safety outcomes including pre-emptively reviewing patients at risk of adverse drug events. This depended upon engagement with the system, the flow of information between different health professionals centrally and locally.  **S2914 –** Using the system could lead to improved medication safety, but use was determined by broad institutional contexts; by the perceptions, dispositions and skills of users; and by the structures embedded within the technology. These included perceptions of the system as new and requiring technical competence and skill; the adoption of the system for information gathering; and interactions and relationships that involved individual, shared or collective use. | Both 3* |
| **2915**  Mlaver E, Schnipper JL, Boxer RB, Breuer DJ, Gershanik EF, Dykes PC, Massaro AF, Benneyan J, Bates DW, Lehmann LS. User-centered collaborative design and development of an inpatient safety dashboard. The Joint Commission Journal on Quality and Patient Safety. 2017 Dec 1;43(12):676-85.  **S2916**  Bersani K, Fuller TE, Garabedian P, Espares J, Mlaver E, Businger A, Chang F, Boxer RB, Schnock KO, Rozenblum R, Dykes PC. Use, perceived usability, and barriers to implementation of a patient safety dashboard integrated within a vendor EHR. Applied clinical informatics. 2020 Jan;11(1):34. | USA | Hospital care | 2915 - To explore the process of integrating an innovative, interactive safety dashboard for inpatient medical services into work flow and confirm that the tool was usable and acceptable to providers.  S2916 - to describe providers' use and perceived usability of the Patient Safety Dashboard and discuss barriers and facilitators to implementation. | 2915 - Qualitative study (usability testing and surveys)  S2916 - Cluster RCT and mixed methods evaluation | 2915 - Through an iterative participatory design process, we developed a novel Web-based clinical patient safety dashboard. We conducted a weeklong proof-of-concept pilot implementation, the medical team reviewed the patient-level screen for each patient on the unit during rounds to confirm that safety issues were being addressed. We administered a Health Information Technology Usability Evaluation Scale (Health-ITUES) survey to the unit nursing and medical directors, attending clinician, and three residents after this pilot implementation. | 2915 - Surveyed users perceived the tool as highly usable and useful. Integration of the dashboard into clinical care is intended to promote communication about patient safety and facilitate identification and management of safety concerns.  S2916 - The Dashboard was used 70% of days the tool was available, with use varying by role, service, and time of day. On general medicine units, nurses logged in throughout the day, with many logins occurring during morning rounds, when not rounding with the care team. Prescribers logged in typically before and after morning rounds. On neurology units, physician assistants accounted for most logins, accessing the Dashboard during daily brief interdisciplinary rounding sessions. Use on oncology units was rare. Satisfaction with the tool was highest for perceived ease of use, with attendings giving the highest rating (4.23) | Both 3* |
| **2918**  Weiss D, Dunn SI, Sprague AE, Fell DB, Grimshaw JM, Darling E, Graham ID, Harrold J, Smith GN, Peterson WE, Reszel J. Effect of a population-level performance dashboard intervention on maternal-newborn outcomes: an interrupted time series study. BMJ quality & safety. 2018 Jun 1;27(6):425-36.  **S2918B**  Reszel J, Dunn SI, Sprague AE, Graham ID, Grimshaw JM, Peterson WE, Ockenden H, Wilding J, Quosdorf A, Darling EK, Fell DB. Use of a maternal newborn audit and feedback system in Ontario: a collective case study. BMJ quality & safety. 2019 Aug 1;28(8):635-44. | Canada | Hospital care | 2918 – To assess the effect of the Maternal Newborn Dashboard on six key clinical performance indicators in the province of Ontario, Canada.  2918B – to increase the understanding of factors explaining variability in performance after implementation of the Dashboard | 2918 – Interrupted time series  2918B – qualitative case study | 2918 – Interrupted time series using population-based data from the provincial birth registry covering a 3-year period before implementation of the Dashboard and 2.5 years after implementation (November 2009 through March 2015).  2918B – A maximum variation sampling approach was used to invite hospitals reflecting different criteria to participate in a 1-day to 2-day site visit by the research team. The visits included: (1) semistructured interviews and focus groups with healthcare providers, leaders and personnel involved in clinical change processes; (2) observations and document review. Interviews and focus groups were audio-recorded and transcribed verbatim. Qualitative content analysis was used to code and categorise the data. | 2918 – Results showed an improvement in four out of six key performance indicators at the provincial level.  2918B – Findings revealed four overarching themes that contribute to the varying success of sites in achieving practice change on the Dashboard key performance indicators, namely, interdisciplinary collaboration and accountability, application of formal change strategies, team trust and use of evidence and data, as well as alignment with organisational priorities and support. The diversity of facilitators and barriers across the 14 hospitals highlights the need to go beyond a ‘one size fits all’ approach when implementing audit and feedback systems. | 2918 – 4* 2918B – 3* |
| **2919**  Dagliati A, Sacchi L, Tibollo V, Cogni G, Teliti M, Martinez-Millana A, Traver V, Segagni D, Posada J, Ottaviano M, Fico G. A dashboard-based system for supporting diabetes care. Journal of the American Medical Informatics Association. 2018 May;25(5):538-47. | Italy | Primary Care | To describe the development, as part of the European Union MOSAIC (Models and Simulation Techniques for Discovering Diabetes Influence Factors) project, of a dashboard-based system for the management of type 2 diabetes and assess its impact on clinical practice. | Mixed methods (before and after and focus groups) | A pre-post study was conducted considering visit duration, number of screening examinations, and lifestyle interventions. A pilot sample of 700 Italian patients was investigated. Judgments on the outcome assessment component were obtained via focus groups with clinicians and health care managers. | The dashboard produced a reduction in visit duration (P ≪ .01) and an increase in the number of screening exams for complications (P < .01). Focus groups highlighted the system’s capability of identifying and understanding the characteristics of patient subgroups treated at the center. | 1* |
| **2920**  Choi HH, Clark J, Jay AK, Filice RW. Minimizing barriers in learning for on-call radiology residents—end-to-end web-based resident feedback system. Journal of digital imaging. 2018 Feb;31(1):117-23. | USA | Hospital care | To minimize barriers to asynchronous communication between attending radiologists and on-call residents, as well as to document quality improvement subjectively through surveys and objectively through participation metrics. | Mixed methods  (surveys and participation metrics) | We assessed quality improvement subjectively through surveys and objectively through participation metrics. | Our web-based feedback system improved user satisfaction for both attending and resident radiologists, and increased attending participation, particularly with regards to cases where substantive discrepancies were identified. | 2* |
| **2921**  Peek N, Gude WT, Keers RN, Williams R, Kontopantelis E, Jeffries M, Phipps DL, Brown B, Avery AJ, Ashcroft DM. Evaluation of a pharmacist-led actionable audit and feedback intervention for improving medication safety in UK primary care: An interrupted time series analysis. PLoS medicine. 2020 Oct 13;17(10):e1003286.  **2921B**  Jeffries M, Gude WT, Keers RN, Phipps DL, Williams R, Kontopantelis E, Brown B, Avery AJ, Peek N, Ashcroft DM. Understanding the utilisation of a novel interactive electronic medication safety dashboard in general practice: a mixed methods study. BMC medical informatics and decision making. 2020 Dec;20:1-4.  **2921C**  Jeffries M, Keers RN, Phipps DL, Williams R, Brown B, Avery AJ, Peek N, Ashcroft DM. Developing a learning health system: insights from a qualitative process evaluation of a pharmacist-led electronic audit and feedback intervention to improve medication safety in primary care. PloS one. 2018 Oct 26;13(10):e0205419. | UK | Primary Care | **2921**  To evaluate changes in the prevalence of hazardous (high-risk) prescribing and inadequate blood-test monitoring associated with this intervention.  **2921B**  To understand the specific ways in which clinical pharmacists and physicians interacted with the SMASH online dashboard and how the level of engagement with the dashboard varied between practices.  **2921C**  To explore the ways in which the intervention was implemented, adopted and embedded into everyday practice (or not) using a qualitative process evaluation | **2921**  Interrupted time series  **2921B**  Mixed methods study  **2921C**  Qualitative process evaluation | **2921**  An interrupted time series analysis of rates (prevalence) of potentially hazardous prescribing and inadequate blood-test monitoring, comparing observed rates post-intervention to extrapolations from a 24-month pre-intervention trend.  **2921B**  Mixed-methods study design involving quantitative data from dashboard user interaction logs from 43 general practices during the first year of receiving the SMASH intervention, and qualitative data from semi-structured interviews with 22 pharmacists and physicians from 18 practices in Salford.  **2921C**  Semi-structured interviews with pharmacists, general practitioners, and other general practice staff | **2921**  Reduced rates of potentially hazardous prescribing and inadequate blood-test monitoring in general practices, sustained over 12 months after the start of the intervention for prescribing but not for monitoring of medication. There was a marked reduction in the variation in rates of hazardous prescribing between practices.  **2921B**  Practices interacted with the dashboard a median of 12.0 (interquartile range, 5.0–15.2) times per month during the first quarter of use to identify and resolve potential medication safety hazards, typically starting with the most prevalent hazards or those they perceived to be most serious. They subsequently worked with GPs to resolve risks on a case-by-case basis, but there were marked variations in processes between some practices. Workload diminished over time as it shifted towards resolving new cases of hazardous prescribing.  **2921C**  Our study highlighted the importance of the combined use of information technology and the role of pharmacists working in general practice settings. Medicine optimisation activities in primary care may be enhanced by the implementation of a pharmacist-led electronic audit and feedback system. | All 3* |
| **2922**  Brown B, Balatsoukas P, Williams R, Sperrin M, Buchan I. Multi-method laboratory user evaluation of an actionable clinical performance information system: Implications for usability and patient safety. Journal of biomedical informatics. 2018 Jan 1;77:62-80.  **2922B**  Tsang JY, Brown B, Peek N, Campbell S, Blakeman T. Mixed methods evaluation of a computerised audit and feedback dashboard to improve patient safety through targeting acute kidney injury (AKI) in primary care. Int J Med Inform. 2020 | UK | Primary care | **2922**  (1) Use a combination of quantitative and qualitative methods to evaluate the usability of PINGR with target end-users; (2) refine existing design recommendations for e-A&F systems; (3) determine the implications of these recommendations for patient safety.  **2922B**  To implement and evaluate an audit and feedback dashboard targeting AKI to improve patient safety, focusing on factors affecting a range of user characteristics in primary care. | **2922**  Mixed methods usability evaluation  **2922B**  Mixed methods study | **2922**  We recruited seven primary care physicians to perform seven tasks with PINGR, during which we measured on-screen behaviour and eye movements. Participants subsequently completed usability questionnaires, and were interviewed in-depth. Data were integrated to: gain a more complete understanding of usability issues; enhance and explain each other’s findings; and triangulate results to increase validity.  **2922B**  Semi-structured interviews were initially performed with both primary (n = 10) and Hospital care (n = 5) staff to gather user requirements for six quality indicators extracted from national guidance on post-discharge AKI care. Modified indicators were implemented in dashboard for six months, across 45 general practices in Salford. Primary care professionals were then interviewed again (n = 7) and completed usability questionnaires. This was triangulated with an interrupted time series analysis on indicator performance, alongside software usage statistics. | **2922**  Participants committed a median of 10 errors (range 8–21) when using PINGR’s interface, and completed a median of five out of seven tasks (range 4–7). Eye movement analysis revealed the integration of components largely supported effective user workflow, although the modular design of clinical performance summaries unnecessarily increased cognitive load. Interviews and questionnaires revealed PINGR is user-friendly, and that improved information prioritisation could further promote useful user action.  **2922B**  Improvements were observed for two out of six quality indicators. A key facilitator to engagement was the development of 'champions of change', achieved through a raised awareness of high-risk patients, guidelines, inconsistencies in coding practice and evidence for quality and safety performance. Barriers related to the specificity and perceived achievability of indicators, and limitations in resources. | Both 3* |
| **2923**  Patel S, Rajkomar A, Harrison JD, Prasad PA, Valencia V, Ranji SR, Mourad M. Next-generation audit and feedback for inpatient quality improvement using electronic health record data: a cluster randomised controlled trial. BMJ Quality & Safety. 2018 Sep 1;27(9):691-9. | USA | Hospital care | To combine known best practices of audit and feedback with newly available continuously generated EHR data to create a modern form of team-based audit and feedback with the goal of improving quality of individual patient care | Cluster randomised controlled trial | Intensive audit and feedback compared with usual audit and feedback from February 2016 to June 2016. Teams in the intensive feedback arm received access to a daily-updated team-based data dashboard as well as weekly in-person review of performance data (‘STAT rounds’). The primary outcome was performance on a composite discharge metric. A washout period occurred at the end of the trial during which STAT rounds were removed from the intensive feedback arm. | Intensive audit and feedback using timely data and STAT rounds significantly increased performance on a composite discharge metric compared with usual feedback. With the cessation of STAT rounds, performance between the intensive and usual feedback groups did not differ significantly, highlighting the importance of feedback delivery on effecting change. | 3* |
| **2950**  Shen X, Lu M, Feng R, Cheng J, Chai J, Xie M, Dong X, Jiang T, Wang D. Web-Based Just-in-Time information and feedback on antibiotic use for village doctors in rural Anhui, China: randomized controlled trial. Journal of medical Internet research. 2018;20(2):e53. | China | Primary care | To test an intervention that provided just-in-time information and feedback (JITIF) to village doctors on care of common infectious diseases. | Randomised controlled trial | 24 village clinics randomized into equal control and intervention arms. Measures used included changes between baseline and endpoint (1 year after baseline) in terms of: percentages of patients with symptomatic respiratory or gastrointestinal tract infections (RTIs or GTIs) being prescribed antibiotics, delivery of essential service procedures, and patients’ beliefs and knowledge about antibiotics and infection prevention. | JITIF was effective in controlling antibiotics prescription at least in the short term and resulted in substantial improvement in delivery of essential service procedures, beliefs favoring rational antibiotics use and knowledge about side effects of antibiotics. | 3* |
| **2992**  Mottes TA, Goldstein SL, Basu RK. Process based quality improvement using a continuous renal replacement therapy dashboard. BMC nephrology. 2019 Dec;20(1):1-0. | USA | Hospital care | To determine if a methodology to assess the quality of CRRT delivery could lead to improvement in CRRT delivery outcomes | Case study | We developed three broad categories of objective CRRT metrics to assess longitudinally, enabling creation of a CRRT Dashboard. Following the objective categories of “filter”, “prescription”, and “fluid balance” over time allowed us to perform quarterly analyses, target provider based CRRT education, and address variation from our standard of care. | We believe our standard for CRRT process measurement will facilitate the creation of standards, quality of care, and best practice guidelines by which benchmarking can be enabled – both within an institution and between institutions. The care for patients receiving CRRT can subsequently improve. | 2* |
| **2994**  Twohig PA, Rivington JR, Gunzler D, Daprano J, Margolius D. Clinician dashboard views and improvement in preventative health outcome measures: a retrospective analysis. BMC health services research. 2019 Dec;19(1):1-3. | USA | Primary Care | To determine if a higher number of dashboard views would be positively associated with clinical quality improvement. | Cross sectional study | A retrospective analysis of change in quality scores compared to number of dashboard views for each PCP over a five-month period | We found no significant correlation between views and change in quality scores for colorectal cancer screening rates and diabetic patients | 2* |
| **2997**  Hester G, Lang T, Madsen L, Tambyraja R, Zenker P. Timely data for targeted quality improvement interventions: use of a visual analytics dashboard for bronchiolitis. Applied clinical informatics. 2019 Jan;10(1):168. | USA | Hospital care | To describe the use of a dashboard in the context of a multifaceted intervention aimed at reducing the use of chest radiographs, bronchodilators, antibiotics, steroids, and viral testing in patients with bronchiolitis. | Case study | Patients 2 months to 2 years old with a bronchiolitis emergency department (ED)/inpatient encounter in the period October 1, 2014 to April 30, 2018 were included. The primary outcome was a functioning dashboard; a process measure was the percentage of ED clinician logins. Outcome measures included the percent use of guideline metrics (e.g., bronchodilators) displayed on statistical process control charts (ED vs. inpatient). Balancing measures included length of stay, charge ratios, and hospital revisits. | In total, 35% of ED clinicians logged in. Leaders used the dashboard to target and track interventions such as a bronchodilator order alert. There were improvements in most outcome metrics; however, timing did not suggest direct dashboard impact. ED balancing measures were lower after implementation. | 2* |
| **2999**  Wu DT, Vennemeyer S, Brown K, Revalee J, Murdock P, Salomone S, France A, Clarke-Myers K, Hanke SP. Usability testing of an interactive dashboard for surgical quality improvement in a large congenital heart center. Applied clinical informatics. 2019 Oct;10(05):859-69. | USA | Hospital care | To evaluate the usability of an interactive surgical dashboard and to seek opportunities for improvement | Mixed methods study | A mixed-method, two-phase study was conducted to collect user feedback. A group of designers (N = 3) and a purposeful sample of users (N = 12) were recruited. The qualitative data were analyzed thematically. The dashboard was evaluated using the System Usability Scale (SUS) and qualitative data. | The participating users gave an average SUS score of 82.9 on the new dashboard. High usability may come from the easy navigation and ability to perform a drill-down analysis. However, the new features also introduced usability issues although these features were designed to fix the user problems identified by the design team in the existing dashboard. | 2* |
| **3003**  Fischer MJ, Kourany WM, Sovern K, Forrester K, Griffin C, Lightner N, Loftus S, Murphy K, Roth G, Palevsky PM, Crowley ST. Development, implementation and user experience of the Veterans Health Administration (VHA) dialysis dashboard. BMC nephrology. 2020 Dec;21:1-2. | USA | Hospital care | To characterize the development, implementation and user experience with Veterans Health Administration (VHA) dialysis dashboard | Qualitative study | A clinical-quality dialysis dashboard was implemented, which displays clinical performance measures (CPMs) for Veterans with ESRD receiving chronic hemodialysis at all VHA facilities. Data on user experience and perceptions were collected via an e-mail questionnaire to dialysis medical directors and nurse managers at these facilities. | Most users of the VHA dialysis dashboard found it accurate, up-to-date, easy to use, and helpful in improving patient care. It meets diverse user needs, including administrative reporting, clinical benchmarking and decision-making, and quality assurance and performance improvement (QAPI) activities. | 2* |
| **3171**  Smalley CM, Willner MA, Muir MR, Meldon SW, Borden BL, Delgado FJ, Fertel BS. Electronic medical record-based interventions to encourage opioid prescribing best practices in the emergency department. The American journal of emergency medicine. 2020 Aug 1;38(8):1647-51. | USA | Hospital care | To measure the effects of using the electronic medical record (EMR) with direct clinician feedback to standardize opioid prescribing practices within a large healthcare system | Before and after study | This retrospective multicenter study compared a 12 month pre- and post-intervention in 14 emergency departments after four interventions utilizing the EMR. Outlying clinicians received feedback through email and direct counselling. Total number of opioid prescriptions per 100 discharges pre- and post-intervention were recorded as primary outcome. | Percentage of total number opioid prescriptions per 100 discharges decreased from 14.4% to 7.4%, a 7.0% absolute reduction, (95% CI,6.9%–7.2%). There was a 5.9% to 0.7% reduction in prescriptions exceeding 3-days, (95% CI, 5.1%–5.3%) and a 0.3% to 0.1% reduction in non-formulary prescriptions, (95% CI, 0.2%–0.3%). | 3* |
| **3202**  Rattray NA, Damush TM, Miech EJ, Homoya B, Myers LJ, Penney LS, Ferguson J, Giacherio B, Kumar M, Bravata DM. Empowering implementation teams with a learning health system approach: leveraging data to improve quality of care for transient ischemic attack. Journal of general internal medicine. 2020 Nov;35(2):823-31.  **3202B**  Bravata DM, Myers LJ, Perkins AJ, Zhang Y, Miech EJ, Rattray NA, Penney LS, Levine D, Sico JJ, Cheng EM, Damush TM. Assessment of the Protocol-Guided Rapid Evaluation of Veterans Experiencing New Transient Neurological Symptoms (PREVENT) program for improving quality of care for transient ischemic attack: a nonrandomized cluster trial. JAMA network open. 2020 Sep 1;3(9):e2015920-. | USA | Hospital care | **3202**  To identify how a virtual “Hub” dashboard that provided performance data for patients with transient ischemic attack (TIA), a resource library, and a forum for sharing QI plans and tools supported QI activities among newly formed multidisciplinary clinical teams  **3202B**  To assess the performance of the Protocol-Guided Rapid Evaluation of Veterans Experiencing New Transient Neurological Symptoms (PREVENT) intervention to improve TIA quality of care. | **3202**  Qualitative evaluation study  **3202B**  Nonrandomized Cluster Trial | **3202**  Qualitative implementation process and summative evaluation of observational Hub data (interviews with Hub users, structured field notes) to identify emergent, contextual themes and patterns of Hub usage.  **3202B**  Nonrandomized cluster trial with matched controls evaluated a multicomponent intervention to improve TIA quality of care at 6 diverse medical centers in 6 geographically diverse states in the US and assessed change over time in quality of care among 36 matched control sites (6 control sites matched to each PREVENT site on TIA patient volume, facility complexity, and quality of care). | **3202**  The Hub supported newly formed multidisciplinary teams in implementing QI plans in three main ways: as an information interface for integrated monitoring of TIA performance; as a repository used by local teams and facility champions; and as a tool for team activation. The Hub enabled access to data that were previously inaccessible and unavailable and integrated that data with benchmark and scientific evidence to serve as a common data infrastructure.  **3202B**  Six facilities implemented the PREVENT QI intervention, and 36 facilities were identified as matched control sites. The implementation of this multifaceted program was associated with improved TIA quality of care across the participating sites. | Both 3* |
| **3578**  Sheen YJ, Huang CC, Huang SC, Lin CH, Lee IT, Sheu WH. Electronic dashboard‐based remote glycemic management program reduces length of stay and readmission rate among hospitalized adults. Journal of Diabetes Investigation. Dec 2020  **s3578b**  Sheen YJ, Huang CC, Huang SC, Huang MD, Lin CH, Lin SY, Sheu WH. Implementation of an electronic dashboard with a remote management system to improve glycemic management among hospitalized adults. Endocrine Practice. 2020 Feb 1;26(2):179-91. | Taiwan | Hospital care  (Single centre) | **3578**  To investigate the impact of a 4-year hospital-wide remote glycemic management program on length of stay  **3578b**  To develop and evaluate the impact of a dynamic dashboard with a remote management system on inpatient glycaemic control | **3578**  Longitudinal observational study  (multi time point)  **3578b**  Longitudinal observational study  (multi time point) | **3578**  In this retrospective study, hospitalized patients who received glucose monitoring were classified into groups 1 (high glucose variability), 2 (hypoglycemia), 3 (hyperglycemia) and 4 (relatively stable). The monthly percentage changes, and average monthly percentage changes of hyperglycemia, hypoglycemia and treat to target were determined using joinpoint regression analysis.  **3578b**  We evaluated the impact of a dynamic dashboard system, which analyzed and monitored all glucose data with virtual glycemic management recommendation by a team of endocrinologists, over 3 × 1-year periods | **3578**  Improved glycemic control was achieved through a hospital-wide electronic remote glycemic management system and reduced LOS and 30-day readmission rates.  **3578b**  After implementation of the dashboard system, the proportion of patients with poor glycemic control (hyperglycemia or hypoglycemia) was reduced by 31% (from 10.2 to 7.0 per day per 100 patients with glucose monitoring; P<.001); hyperglycemia decreased by 25% (from 6.1 to 4.6 per day per 100 patients with glucose monitoring; P<.001), and hypoglycemia decreased by 45% (from 4.2 to 2.3 per day per 100 patients with glucose monitoring; P<.001). | Both 2* |
