# Supplementary material for: Systematic review and narrative synthesis of computerized audit and feedback systems in healthcare
Source: J Am Med Inform Assoc. 2022 Mar 10;29(6):1106–19. doi: 10.1093/jamia/ocac031 (PMC9093027; doi:10.1093/jamia/ocac031)
Supplement: ocac031_Supplementary_Data [file ocac031_supplementary_data.zip › Additional file 4 (Best prac graph).docx]

# Additional file 4: Graph illustrating the number of best practices (out of 18) adopted by systems over time


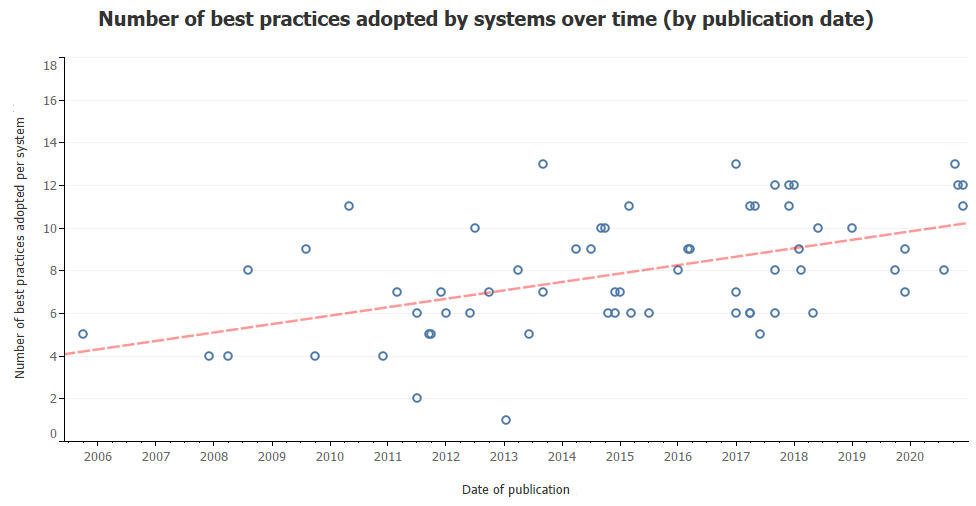


The scatter graph above illustrates the number of best practice features (out of 18) that were adopted by computerised audit and feedback (e-A&F) systems over time – represented by the blue line. The pink line illustrates the linear regression line, showing an estimated increase of 0.40 (95% confidence intervals 0.32-0.48) best practice features per year, though heterogeneity was observed between systems.
