# Supplementary material for: Systematic review and narrative synthesis of computerized audit and feedback systems in healthcare
Source: J Am Med Inform Assoc. 2022 Mar 10;29(6):1106–19. doi: 10.1093/jamia/ocac031 (PMC9093027; doi:10.1093/jamia/ocac031)
Supplement: ocac031_Supplementary_Data [file ocac031_supplementary_data.zip › Additional file 5 (ICMO, CERQUAL).docx]

# Additional file 5: Full list of hypotheses and GRADE-CERQual ratings

The following table presents our GRADE-CERQual (Lewin et al.) ratings for each Intervention-context-mechanism-outcome (ICMO) configuration noted within our review. Each is rated with ‘high’, ‘moderate’, or ‘low’ confidence of the finding within our synthesis through assessing methodological limitations, relevance, coherence and adequacy of data, particularly focussing on the evidence supporting that review finding, and how the review finding relates to the wider review question.

*S. Lewin, C. Glenton, H. Munthe-Kaas, B. et al., Using Qualitative Evidence in Decision Making for Health and Social Interventions: An Approach to Assess Confidence in Findings from Qualitative Evidence Syntheses (GRADE-CERQual), PLOS Med. 12 (2015) e1001895. doi:10.1371/journal.pmed.1001895.*

| Intervention* | Context* | Mechanism* | Outcome* |
| --- | --- | --- | --- |
| *Timely* feedback – supported by availability of **near real-time feedback** | Supportive *organisational* *context*  *A&F attitude* positive | Enabled *actionability* (Pre-requisite)  *Perceived relative advantage*  Higher *credibility*  Higher *compatibility* | Higher engagement with *feedback cycle*  More likely to lead to clinical improvements |
| References: 21,22,24,26,27,31,33,39,41,44,52,55,57,58,66,70,71,75,76,80,82,83,86–88,90–92,94,97,101,102,105–107  GRADE- CERQUAL rating: High | | | |
| Less *timely* feedback – though still **quicker** compared to manual audit and previous systems | Supportive *organisational context* | Retains *perceived relative advantage*.  Limited *actionability*  *Incompatibility with existing practices* | Unable to *act* – time of action/decision making already past  Non-engagement – drop out |
| References: 23,37, 38,40,42,48–50,53,54, 61,73,85,98,99,108  GRADE- CERQUAL rating: High | | | |
| *Patient lists* for **frontline users**  At least *team* or *practice* level feedback | *Intra-organisational networks* (particularly for *primary care*)  *A&F attitude* positive | Increased *actionability*  Increased *credibility* - can scrutinise feedback of patient lists  Lower *complexity* of task | Enabled concrete *actions* (‘working through the list’)  Increased individual *ownership*  More likely to lead to clinical improvements |
| References: 21,24,26–30,41,48,49,52,55,57,58,60,63,66,71,74–76,78,80–83,90–92,94,97,104,106,107  GRADE- CERQUAL rating: High | | | |
| *Individual performance feedback* for **managerial users**  Violating the underlying *assumption* that good care is being provided | Strong *leadership support*  *Intra-organisational networks*  +/- *Champions* | Positive *social influence*  Increased *credibility*  Increased *actionability* | Increased individual *ownership* of feedback  Positive influence on *feedback cycle*  More likely to lead to clinical improvements |
| References: 22,25,27,31,32,35,37,40,42,43,48,50,51,53,54,56,59–61,63,65,67,69,72–74,77,79,84–86,89,91–97,99,100,103,104,107  GRADE- CERQUAL rating: High | | | |
| **User role-specific** *performance level feedback* to highlighting gaps in care, particularly through concise *visualisations*, *prioritisation* features (e.g. *colour coding*, *sorting* functions) | *A&F attitude* positive  Supportive *organisational context* | Increased a*ctionability*  Increased *problem solving*  Lower *complexity* of the task  Increased *credibility* of feedback | Positively influences *feedback cycle*.  More likely to produce clinical improvements. |
| References: 21,26,27,35,39,41,43,48,53–55,57,60,65,69,70,72,74,75,77–81,85,86,91,92,95,100–103,107  GRADE- CERQUAL rating: Moderate (Some concerns regarding relevance) | | | |
| Multiple *graphical elements* of performance data | *A&F attitude* positive  Supportive *organisational context* | Increased *problem solving*  Increased *credibility* of feedback  Can both increase or decrease *complexity* | Positively influences *feedback cycle*.  Too many *graphical elements* can overload users and decrease *readability*  Some *graphical elements* too complex  Some *perception* of unmanageable workload. |
| References: 21,22,24–28,31,33,37,40,42,43,48,50,51,53–57,59,60,67,69,72–75,77–81,84–86,89,91,93,94,96,101–103,107  GRADE- CERQUAL rating: Low (Some concerns regarding methodological limitations and major concerns regarding relevance) | | | |
| No **user role-specific** feedback  *Benchmarking*  *Peer discussion*  Triggering *reflections* to *reinforce change*  +/- *Feedback delivered to a group* | *Leadership support*  *Champions*  *intra-organisational networks*  *Financial rewards or alignment* | Limited *actionability*  Positive *social influence*  Positive *resource match* | Enabled better implementation and higher engagement in *feedback cycle*  Increased *ownership* of feedback by frontline users  Insufficient by itself to support e-A&F to produce clinical improvements |
| References: 21,22,24,25,27,28,31–33,37,39,40,42,43,48,50–57,59,60,62,63,65,67,69,73–75,77–81,84–86,89,91–93,95–97,99–101,103–105,107  GRADE- CERQUAL rating: Moderate (Some concerns regarding methodological limitations and regarding coherence of findings) | | | |
| eA&F focussing on **measurement**  *Summarising data*  Facilitating *surveillance* | Generally supportive *organisational context*  *Extra-organisational networks*  Limited *adaptation* for local buy in | Perceived *relative advantage* to help improve care.  Reduced *complexity* of the task  Limited ac*tiona*bility  Some concerns regarding *credibility* and *compatibility* | Higher engagement in *feedback cycle*, due to raised awareness and increased attention to task, but alone was insufficient to produce clinical improvements.  Limited *ownership* of feedback by frontline users.  Some concerns regarding *punitive feedback*  Some concerns about the limitations of feedback |
| References: 23,27,37,38,42,48,49,54,57,63,65,68,77,79,82,83,85,89,99–101,105,106,108  GRADE- CERQUAL rating: High | | | |
| *Delivery of feedback to more junior staff*  *Benchmarking* | Supportive *organisational context* | Positive *social influence*  *Resource match*  Some concerns regarding *credibility* and *compatibility* | Higher engagement in *feedback cycle* as some junior staff more receptive to feedback  Some *junior staff* have more *capacity* and flexibility for learning and development  Some concerns regarding *credibility*, as performance frequently affected by *senior influence* or actions  Some issues with *ownership* of feedback |
| References: 24,33,44,63,64,66,75,76,89,93,97  GRADE- CERQUAL rating: Moderate (Moderate concerns regarding coherence and relevance of findings. | | | |
| *Action plans* **embedded** within e-A&F system | *A&F attitude* positive | Increased *actionability*  Increased *problem solving*  Lower *complexity* of the task | Positive influence on *feedback cycle* (especially *goal setting*)  More likely to lead to clinical improvements |
| References: 24,33,35,36,44,55,56,62,66,73,75,76,79,84,91  GRADE- CERQUAL rating: High | | | |
| *Action plans* not **embedded** within e-A&F system | Despite:  *Intra-organisational networks*  *Extra-organisational networks*  *Leadership support* | Limited *actionability*  Limited *problem solving*  Increased *complexity* | Seen as ‘extra-step’ or additional work  Some ‘unrealistic’ action plans and ‘unattainable’ goals created |
| References: 23,25,32,34,42,43,45–47,54,72,99,101,108  GRADE- CERQUAL rating: High | | | |
| Lack of *action plans* | No specific *organisational support* or barriers | Limited *actionability*  Limited *problem solving*  Increased *complexity* | Lower engagement and usage  e-A&F systems not achieving full effects. |
| References: 42,77,89,93  GRADE- CERQUAL rating: Low (Major concerns regarding methodological limitations, relevance and adequacy) | | | |

*Constructs taken from clinical performance feedback intervention theory (CP-FIT) are in *italics*, with nuanced codes that were added inductively in **bold.**
