# Supplementary material for: Systematic review and narrative synthesis of computerized audit and feedback systems in healthcare
Source: J Am Med Inform Assoc. 2022 Mar 10;29(6):1106–19. doi: 10.1093/jamia/ocac031 (PMC9093027; doi:10.1093/jamia/ocac031)
Supplement: ocac031_Supplementary_Data [file ocac031_supplementary_data.zip › Additional file 6 (Mechanisms).docx]

# Additional file 6: Descriptions and explanations of Mechanism constructs

The following table presents the descriptions and explanations of the mechanisms constructs used within our results tables, consistent with Clinical Performance Feedback Intervention Theory (CP-FIT) by Brown et al. 2019.

*Brown B, Gude WT, Blakeman T, et al. Clinical Performance Feedback Intervention Theory (CP-FIT): a new theory for designing, implementing, and evaluating feedback in health care based on a systematic review and meta-synthesis of qualitative research. Implement Sci. 2019;14(1):40.*

| **Mechanism construct** | **Descriptions and Explanations** |
| --- | --- |
| Actionability^$^ | ***Description***: The ability of e-A&F systems to directly facilitate behaviours for users, inducing changes by contributing to a tangible next step or concrete action to improve patient care.  ***Explanation***: The more an e-A&F system can successfully and directly support clinical behaviours with tangible or concrete next steps, the more users are empowered and motivated to act these behaviours, also increasing perceived achievability and controllability of the task. |
| Relative advantage | ***Description***: Recipients’ perceived benefits of the e-A&F systems, often when compared to alternative existing or proposed ways of working. Aspects of e-A&F systems considered to have a relative advantage are (understandably) situation-specific, so its appearance as a mediating variable is inconsistent.  ***Explanation***: The more an e-A&F system has a perceived advantage over current ways of working, the more likely it is to be adopted within the available resources. |
| Credibility | ***Description***: The perceived trustworthiness and reliability of the e-A&F system.  ***Explanation***: the more trustworthy and reliable an e-A&F system, the more likely they are to believe it will help them improve patient care. |
| Compatibility | ***Description***: The degree of ‘fit’ between the e-A&F system and characteristics of the recipient and their organisation e.g. beliefs, norms, values, culture, structures, processes, technical systems.  ***Explanation***: the more an e-A&F system can align with the beliefs, systems, and processes of an organisation and its staff, the greater its relevance, and the less disruption required for its implementation. |
| Complexity | ***Description***: The difficulty of performing the e-A&F system processes and tasks.  ***Explanation***: Due to capacity limitations of users and organisations to engage with feedback, the simpler a system is to engage with, the less resource it requires. |
| Social influence | ***Description***: Interpersonal processes that cause e-A&F system users to change their thoughts, feelings, or behaviours. Key aspects include: 1) Competition (between health professionals), 2) Social proof (their desire to behave in the same way as other health professionals), 3) Authority (to obey credible authority figures), 4) Liking (persuaded by people they like), and 6) Reference group (where health professionals feel part of a group and will change their behaviour if they believe their membership of that group is threatened).  ***Explanation***: the more an e-A&F system can harness the social dynamics between health professionals, the more likely it is to be implemented. |
| Problem solving^$^ | ***Description:*** Analysis of reasons for sub-optimal clinical performance and formulation of solution(s) to address them. This may be performed as part of the e-A&F system, or the recipients may be required/supported to do it themselves e.g. via peer discussion, support from champions or through an action plan.  ***Explanation***: Increases motivations and behaviours through providing practical support on how to act effectively to the feedback message and addressing health professionals’ lack of knowledge and skills to perform these behaviours. |
| Resource match | ***Description***: Whether the associated costs of the intervention are matched by the available resource.  ***Explanation***: If a healthcare organisation’s resources match the costs of implementing an e-A&F system they are more likely to engage and respond to it. |

^$^The descriptions and explanations for these codes have been further nuanced compared to the original code in CP-FIT.
